# Nanoencapsulation of *Rhodiola rosea* extract into 2-hydroxypropyl-β-cyclodextrin: enhanced antibacterial and anticancer activities

**DOI:** 10.1039/d5ra07949g

**Published:** 2026-02-26

**Authors:** Shaza Khorshed, Ahmed Maher Abdeldayem, Wolfgang Fritsche, Hassan Mohamed El-Said Azzazy

**Affiliations:** a Department of Chemistry, School of Sciences & Engineering, The American University in Cairo New Cairo 11835 Egypt hazzazy@aucegypt.edu; b Department of Nanobiophotonics, Leibniz Institute of Photonic Technology Jena 07745 Germany

## Abstract

The methanolic extract of *Rhodiola rosea* (RRME) exhibits multifaceted biological functions, notably antioxidant, anti-inflammatory, and neuroprotective potential. These properties are primarily associated with its rich therapeutic phytochemicals such as salidroside, rosavin, and tyrosol, which contribute to its potential therapeutic applications in managing stress-related disorders and neurodegenerative conditions. In this study, RRME was extracted using the Soxhlet method and subsequently encapsulated in (2-hydroxypropyl)-beta-cyclodextrin (HPβCD) using the freeze-drying technique, forming inclusion complexes (RRME-ICs). RRME-ICs were spherical morphologies with a size range of 87.36 to 166.5 nm. The ICs had a polydispersity index of 0.283 ± 0.01 and %EE of 78.88 ± 1.55%. FT-IR, ^1^H NMR, 2D NMR NOESY, and TGA demonstrated the effective incorporation of RRME into HPβCD and enhanced integrity with a high thermal stability up to 350 °C. RRME-ICs demonstrated strong antibacterial effects compared to the extract. The MIC90 of RRME exceeded 20 mg mL^−1^ against *Escherichia coli*, *Pseudomonas putida*, and *Staphylococcus aureus*, indicating limited antibacterial potency. Release kinetics analysis confirmed that the Korsmeyer–Peppas model provided the best fit for RRME-ICs across both pH conditions. RRME-ICs demonstrated remarkable antibacterial activity, with IC_90_ of 2.38, 3.95, and 1.92 mg mL^−1^ against *E. coli*, *P. putida*, and *S. aureus*, respectively. Additionally, RRME-ICs preserved antioxidant activity in the DPPH scavenging assay, demonstrating 92.6% scavenging efficiency with an IC_50_ value of 0.0657 mg mL^−1^. RRME-ICs also exhibited notable anticancer potential against melanoma (A375) cells with IC_50_ of 48.14 µg mL^−1^, compared to RRME (IC50 = 131.24 µg mL^−1^). In conclusion, encapsulation through HPβCD inclusion complex formation improved its antibacterial and anticancer activities while preserving its antioxidant activity.

## Abstract 2

Encapsulation of *Rhodiola rosea* methanolic extract into HPβCD enhances its cytotoxicity against human melanoma (A375) cells and its antimicrobial activity against *Pseudomonas putida*, *Escherichia coli*, and *Staphylococcus aureus*.

## Graphical abstract

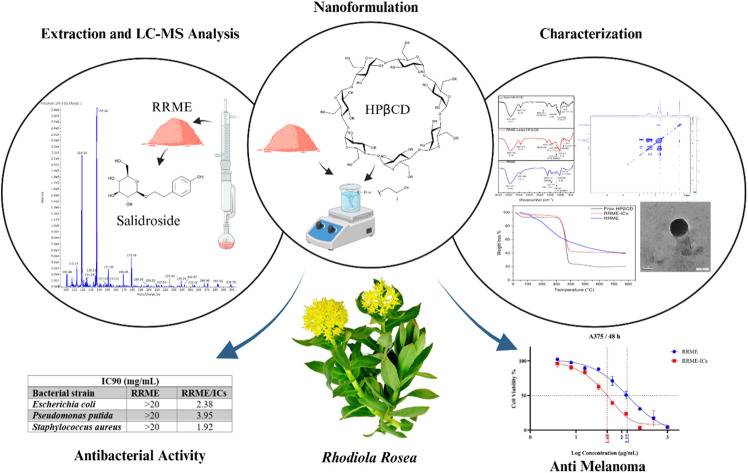

## 1 Introduction

**Fig. 1:**
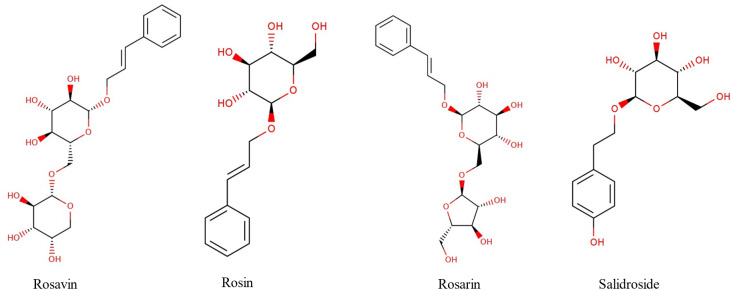
Chemical structures of bioactive compounds of *Rhodiola rosea*.

*Rhodiola rosea* (RR) is a member of the Crassulaceae family, also termed Roseroot, Golden Root, and Arctic Root. RR originated in Asia (Siberia), North America, and Britain. The *Rhodiola genus* contains about 200 species, including *R. kirilovii*, *R. crenulata*, *R. sacra*, *R. alterna*, *R. quadrifia*, and *R. rosea*. *R. rosea* extract was shown to be useful in treating chronic diseases.^1^ The chemical profile of RR extract includes several phytoconstituents as phenolic acids, flavonoids, and tannins. It contains *trans*-cinnamic acid derivatives including rosavin, rosin, and rosarin, which are known as “Rosavins”. These compounds (Fig. 1) distinguish it from other *Rhodiola* species.^1–4^ Additionally, phenylethanoids, including salidroside, tyrosol derivatives, and *p*-tyrosol, represent the second major class of bioactive constituents in *Rhodiola rosea*.^1^

Extraction of *Rhodiola rosea* using methanol and ethanol-based Soxhlet extraction yielded extracts enriched with bioactive compounds that exhibit potent antioxidant, antibacterial, and anticancer activities.^5^ Despite its promising pharmacological profile, similar to other plants, *Rhodiola rosea* extract has low solubility, chemical instability, and limited absorption, which may hinder its optimal therapeutic potential.^6^

Enhancing the therapeutic efficacy of drugs and natural extracts with low bioavailability and stability remains a challenge in pharmaceutical development. One promising strategy involves nanoencapsulation in nanosystems including β-cyclodextrin (βCD). βCD is structurally defined by a circular arrangement of oligosaccharide, presenting a hydrophilic exterior and a hydrophobic interior, which enables it to encapsulate hydrophilic as well as hydrophobic molecules. β-Cyclodextrin (βCD) has several modified forms such as hydroxypropyl-βCD (HPβCD), methylated-βCD (MβCD), sulfobutylether-βCD (SBEβCD), carboxymethyl-βCD (CMβCD), and acetylated βCD which exhibit improved aqueous solubility, tunable polarity, and functional versatility tailored to specific pharmaceutical and analytical applications. These structural modifications influence guest affinity, toxicity profiles, and release kinetics, making them valuable tools in drug delivery and molecular encapsulation systems. Additionally, they protect bioactive compounds against degradation, thereby enhancing their pharmacokinetic properties.^7–11^ Beyond these physicochemical advantages, βCD-based carriers are designed to minimize dosing frequency by enabling prolonged and predictable drug release profiles. Incorporation of βCD-functionalized nanoparticles facilitates this release behavior, potentially lowering the required therapeutic dose.^12^ Velu *et al.* (2024) demonstrated that β-cyclodextrin–nitazoxanide inclusion complexes significantly improved NTX solubility and *in vitro* release characteristics, indicating potential for reduced dosing frequency by enhancing bioavailability and prolonging intracellular retention.^13^

In this work, the *Rhodiola rosea* methanolic extract (RRME) was prepared, characterized, and encapsulated within 2-hydroxypropyl-β-cyclodextrin (HPβCD). Additionally, the antioxidant, antibacterial, and anticancer activities of *Rhodiola rosea* extract were investigated and assessed relative to those of RRME-HPβCD inclusion complexes.

## 2 Materials and methods

### 2.1 Reagents

Methanol (HPLC grade) and 2-hydroxypropyl-β-cyclodextrin were obtained from Sigma-Aldrich (Taufkrichen, Germany). Acetonitrile (HPLC grade) was purchased from VWR BDH Chemicals (Fontenay-sous-Bois, France). KBr (FT-IR grade) and dimethyl sulfoxide were obtained from Merck (KGaA, Darmstadt, Germany). DPPH Powder, Follin Ciocalteu reagent and nutrient broth were obtained from Titan Biotech Ltd (Rajasthan, India). Antibiotics, l-glutamine, and FBS were acquired from Capricorn, (Germany). Resazurin was obtained from SERVA GmbH (Germany).

### 2.2 Extraction of *Rhodiola rosea*

Extraction was performed following a previously published method of Kosakowska *et al.* (2018)^5^ with minor adjustments. Briefly, dried powder (12 g) was weighed and put it in Soxhlet apparatus with 120 mL of methanol in a rounded-bottom flask fixed into a heat mantle at 60 to 70 °C. Following an 8-h extraction period, the mixture was placed in a vacuum oven and dried at 60 °C to facilitate solvent volatilization. The evaporated solvent was subsequently condensed to obtain the methanolic extract solution. The obtained powder was dark brown in color and had a light aroma.

### 2.3 LC-MS analysis

The analysis was conducted, following a previously published protocol,^14^ utilizing the ExionLC AC system for chromatographic separation and the SCIEX Triple Quad 5500+ MS/MS instrument, operated with electrospray ionization (ESI) for compound detection. Minor modifications were introduced to the negative ionization mode during MS/MS analysis to enhance detection sensitivity for the targeted analytes^15–18^

### 2.4 Encapsulation of RRME into HPβCD

The entrapment of RRME within HPβCD was executed through the formation of their inclusion complexes (ICs) using lyophilization as reported previously.^19,20^ Briefly, to prepare the RRME-2-hydroxypropyl beta-cyclodextrin inclusion complexes (RRMR-ICs), 4 g of HPβCD were dispersed in 20 mL of deionized water. Subsequently, 400 mg of RRME were transferred to the solution and subjected on the stirrer (200 rpm) at room temperature for 24 h in a sealed container, isolated from light exposure, to allow the formation of RRME-ICs. The suspension was then passed through a 0.22 µm PTFE membrane to remove any unloaded RRME molecules. The filtered RRME-ICs were stored at −20 °C for approximately 18 h before undergoing lyophilization with a freeze dryer to eliminate residual moisture. Finally, the lyophilized residue was preserved in a tightly closed, light-protected bottle until further use.

### 2.5 Particle size and PDI analysis

The particle size and PDI were assessed using Malvern zetasizer Instruments Ltd, Malvern, UK. To prepare the samples, RRME-ICs and HPβCD powders were individually suspended in distilled H_2_O (4 : 1; w/v).^21^ Measurements were carried out at 25 °C.

### 2.6 High-resolution transmission electron microscopy

Morphological analysis of the encapsulated extract was performed using a JEOL-1010 a high-resolution transmission electron microscope (JEOL, Tokyo, Japan). To enhance contrast and structural visualization, samples were subjected to negative staining prior to analysis. A sample drop was applied to the surface of Cu grids coated with carbon. A drop of phosphotungstic acid solution (2%) was then added and samples were allowed to stain for 15 min before being dried at room temperature. The RRME encapsulated within β-cyclodextrin (β-CD) was adhered to the carbon-coated grid surface, stabilized by a surfactant polymer containing tungsten atoms to enhance absorption contrast.^19–21^

### 2.7 FT-IR analysis

Fourier-transform infrared spectral analysis of HPβCD, RRME, and RRME-ICs was done (4000 cm^−1^ to 400 cm^−1^) to compare the relevant functional groups and assess the encapsulation of RRME into cyclodextrins. Prior to analysis, a small amount of each sample was first homogenized with FT-IR grade KBr powder and subsequently compressed into thin, transparent discs using a hydraulic press to ensure uniformity.^22^

### 2.8 Determination of entrapment efficiency (% EE) and drug loading (% DL) capacity

The quantification of RRME entrapped within the RRME-ICs was performed using a Ultraviolet-visible spectroscopy (Cary3500, Agilent Technologies, Mulgrave, Australia). RRME-ICs (3 mg) was dispersed in acetonitrile (99%, HPLC- and UV-grade) (3 mL) within a covered falcon tube, shielded from light. Subsequently, the container was placed on an orbital shaker and maintained at room temperature for one week, to enable the encapsulated RRME to be released into the acetonitrile solution.^19,20^ Afterwards, a small aliquot of the supernatant solution, containing the released RRME, was withdrawn and absorbance measured at 263 nm.^23^ To construct a calibration curve, a stock solution of RRME was prepared, followed by serial dilutions to generate a series of concentrations from 15.625 to 500 µg mL^−1^. The following equations were utilized to determine the % EE and % DL of the RRME-ICs.
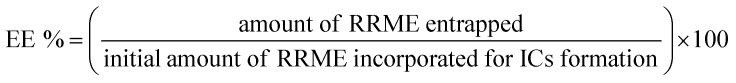

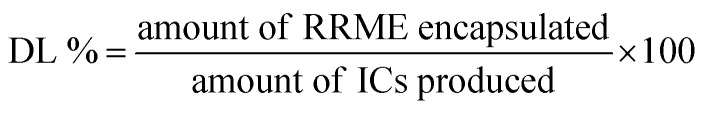


### 2.9 ^1^H and 2D-NOESY NMR spectra

The proton spectra of RRME, HPβCD and RRME-ICs as well as the two-dimensional NOESY of RRME-ICs were performed using an NMR spectrometer (BRUKER BioSpin GmbH, Rheinstetten, Germany) operating at 400 MHz and kept at 25 °C. Samples were dissolved in DMSO and placed into NMR tubes for analysis.

### 2.10 Thermogravimetric analysis (TGA) of the extract, HPβCD and RRME-ICs

TGA was performed to evaluate the thermal integrity and decomposition behavior of the RRME, HPβCD and RRME-ICs. The samples were exposed to a controlled thermal program at a rate of 10 °C min^−1^, progressively increasing the temperature (from ambient up to 800 °C).^19,20,24^

### 2.11 Release profile of RRME-ICs

*In vitro* release profile of RRME-ICs was evaluated using the dialysis bag method^25^ with minor modifications. Briefly, 10 mg of freeze-dried NP formulations were dispersed in 2 mL PBS (pH 7.4 or 5.5) and loaded into sealed dialysis bags (MW cutoff of 3.5 kDa). These were immersed in beakers containing 20 mL of the corresponding release medium and incubated in a shaking incubator (JSSI-100, JSR, Korea) at 37 °C and 100 rpm. At specified intervals (0.25, 0.5, 1, 2, 4, 6, 9, 12, 24, 48, 144, and 168 h), 1 mL aliquots of the release medium were withdrawn and replaced with an equal volume of fresh medium. RRME concentrations in the samples were quantified by UV spectroscopy at 263 nm. All experiments were performed in triplicate.

### 2.12 Release kinetics modeling

To elucidate the kinetics of RRME release from RRME-ICs, the dissolution data were fitted to zero-order, first-order, Higuchi, and Korsmeyer–Peppas models using GraphPad Prism 10.3.0 (507) for windows (San Diego, California USA). The model providing the highest coefficient of determination (*R*^2^) and appropriate release exponent (*n*) was selected as the best fit.^26,27^

The zero-order model assumes a constant release rate independent of concentration and was evaluated by plotting cumulative percentage drug release *versus* time using the following equation:*Q*_*t*_ = *Q*_0_ + *K*_0_*t*where *Q*_*t*_ is the amount of drug released at time *t*, *Q*_0_ is the initial drug content, and *K*_0_ is the zero-order release rate constant.

The first-order model describes concentration-dependent release kinetics and was assessed by plotting the natural logarithm of the percentage drug remaining *versus* time, according to equation:Log *Q*_*t*_ = log *Q*_0_ − *K*_*t*_/2.303where *Q*_*t*_ is the amount of drug remaining (or released) at time *t*, *Q*_0_ is the initial drug amount, *K* is the first-order release rate constant, and 2.303 is the conversion factor for base-10 logarithm.

The Higuchi model, which describes diffusion-controlled release from a matrix, was evaluated by plotting cumulative percentage drug release *versus* the square root of time, according to the equation:*Q*_*t*_ = *K*_*H*_*t*^1/2^where *K*_H_ is the Higuchi rate constant.

The Korsmeyer–Peppas model, used to characterize the release mechanism, was analyzed by plotting log(cumulative percentage drug release) *versus* log *t*, according to the following equation:
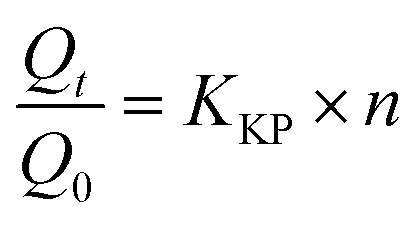
where 
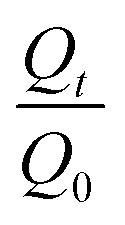
 represents the fraction of drug released at time *t*, *K*_KP_ is the Korsmeyer–Peppas release rate constant, and *n* is the release exponent indicative of the diffusion mechanism.

### 2.13 Folin–Ciocalteu assay

The polyphenolic content of the RRME extract was measured using the Folin–Ciocalteu assay. Gallic acid (an antioxidant standard) was diluted from 3.125 to 500 µg mL^−1^ to generate a standard curve. The test sample (0.1 mL) was dispersed in a test tube, and the volume brought to 0.5 mL with deionized water. Subsequently, Folin–Ciocalteu reagent (0.25 mL) was added, followed by the addition of 1.25 mL of 10% Na_2_CO_3_. The samples were vortexed thoroughly, and the absorbance of the resulting blue mixture determined at 725 nm after incubation (40 min) at room temperature. A blank sample, containing 0.1 mL of the extraction solvent was used as a reference for baseline absorbance.^28^

### 2.14 Radical scavenging assay

The antioxidant potential of RRME was assessed *via* the DPPH (1,1-diphenyl-2-picryl hydrazyl) assay to assess its hydrogen-donating ability and radical scavenging capacity. DPPH solution in methanol (3 mL of a 0.12 mM) was added to 100 µL RRME (1 mg/2 mL methanol). Ascorbic acid, used as standard antioxidant, was prepared in different concentrations (0.03125–0.5 mg mL^−1^) in methanol to generate the standard curve. The absorbance of mixtures was determined at 517 nm and incubated for 30 min, kept at room temperature.^28^ For the blank sample, 1 mL of methanol was used. The radical scavenging capacity of the samples was obtained using the following formula:*I* = [(*A*_B_ − *A*_A_)/*A*_B_] × 100where *I* is scavenging activity (%); *A*_B_ represents absorbance of the blank, while *A*_A_ corresponds to absorbance of RRME measured after 30 min.

The antioxidant activity of RRME was quantified in terms of its IC_50_ value, representing the concentration (mg mL^−1^) at which the RRME exhibits half-maximal inhibition of DPPH radicals.

### 2.15 Antimicrobial activity assay

The minimum inhibitory concentration (MIC) of RRME and RRME-ICs against *Staphylococcus aureus* ATCC 25923 (*S. aureus*), *Escherichia coli* ATCC 25922 (*E. Coli*), and *Pseudomonas putida* ATCC 12633 (*P. putida*) was determined using a microdilution assay. Bacterial broth was prepared by cultivating each strain in nutrient broth overnight. Concentrations of the suspensions were adjusted to 10^6^ CFU mL^−1^. Serial dilutions of RRME and RRME-ICs were applied in a 96-well plate to achieve 0.625, 1.25, 2.5, 5, 10, and 20 mg mL^−1^. Subsequently, the standardized bacterial broth was added to each well. Samples were added using the serial dilution in triplicate for each concentration. The bacterial cultivation was carried out by maintaining the plates at 37 °C for a 24-h period. Then, 50 µL of Alamar Blue reagent (75 µg mL^−1^) were introduced to each well. A color transition from blue to pink evidenced metabolically active bacterial cells, while the elimination of a color transition signified bacterial growth inhibition.^29^

### 2.16 Anti-melanoma activity assay

Human melanoma A375 cells, purchased from Nawah Scientific, Cairo, Egypt, were grown in RPMI-1640 enriched with 10% fetal bovine serum and supplemented with antibiotics, including streptomycin (100 µg mL^−1^) and penicillin G (100 U mL^−1^) and 2 mM l-glutamine. For passaging, monolayer cells were treated with trypsin/EDTA at 37 °C when confluence reached 75%.^30^ Cells (1 × 10^4^ cells per well) were seeded in a flat bottom 96-well microplate and treated with 50 µL of different concentrations of RRME and RRME-ICs (3.906, 7.813, 15.625, 31.25, 62.5, 125, 250, 500 and 1000) and incubated (48 h) at 37 °C in a 5% CO_2_ incubator. Then media were aspirated and wells washed twice by 1X PBS, pH 7.2, then 50 µL of 75 µg mL^−1^ resazurin (Alamar solution) were subjected to each well and incubated for 4 h in a CO_2_ incubator. Fluorescence was measured at 590 nm following excitation at 540 nm (FLUOstar OPTIMA, Ortenberg, Germany).^31^ Cell viability was determined using the equation:
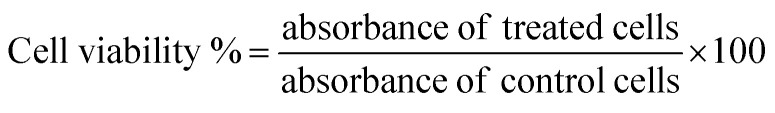


## 3 Statistics

Half maximal inhibitory concentration (IC_50_) and dose response curve were calculated using non-linear regression analysis with Asymmetric (five parameter), *X* is log(concentration) equation using GraphPad Prism 10.3.0 (507) for windows (San Diego, California USA).

## 4 Results and discussion

### 4.1 Extraction of *Rhodiola rosea*

The obtained powder was dark brown in color and had a light aroma. The dark brown color of RRME arises primarily from polyphenolic compounds (including tannins, flavonoids, and phenolic acids) which are abundant in *R. rosea* and correlate with the extract's antioxidant activity observed. Studies on similar herbal extracts confirmed that methanol efficiently solubilizes these colored phenolics,^32^ resulting in yields and coloration consistent with yield and color obtained in this study. Moreover, methanolic maceration yielded 5.6% RRME enriched in salidroside, rosavin, and phenolics consistent with solvent polarity favoring semi-polar glycosides and antioxidants.^33^ In comparison, 70% ethanolic extraction typically results in rosavin dominance but lower phenolics,^34^ while aqueous methods prioritize polysaccharides.^35^ Supercritical CO_2_ extraction, even with methanol modifier, yields volatiles/salidroside but excludes phenolics.^36^ Therefore, methanol extraction yields a balanced profile, enabling synergistic bioactivity across these structurally correlated compounds.

### 4.2 LC-MS analysis

**Fig. 2:**
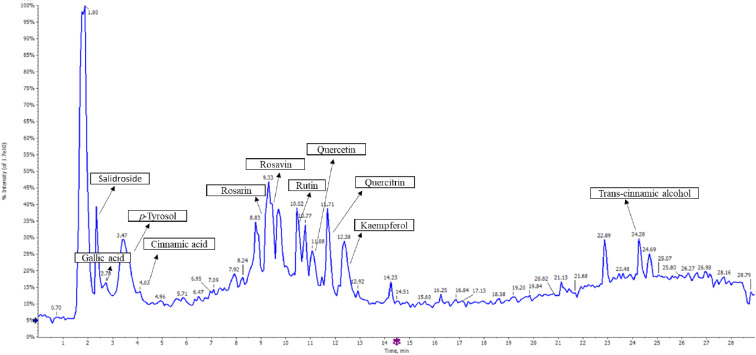
LC-MS chromatogram (intensity % *vs.* retention time) of the methanolic extract of *Rhodiola rosea*.

**Fig. 3:**
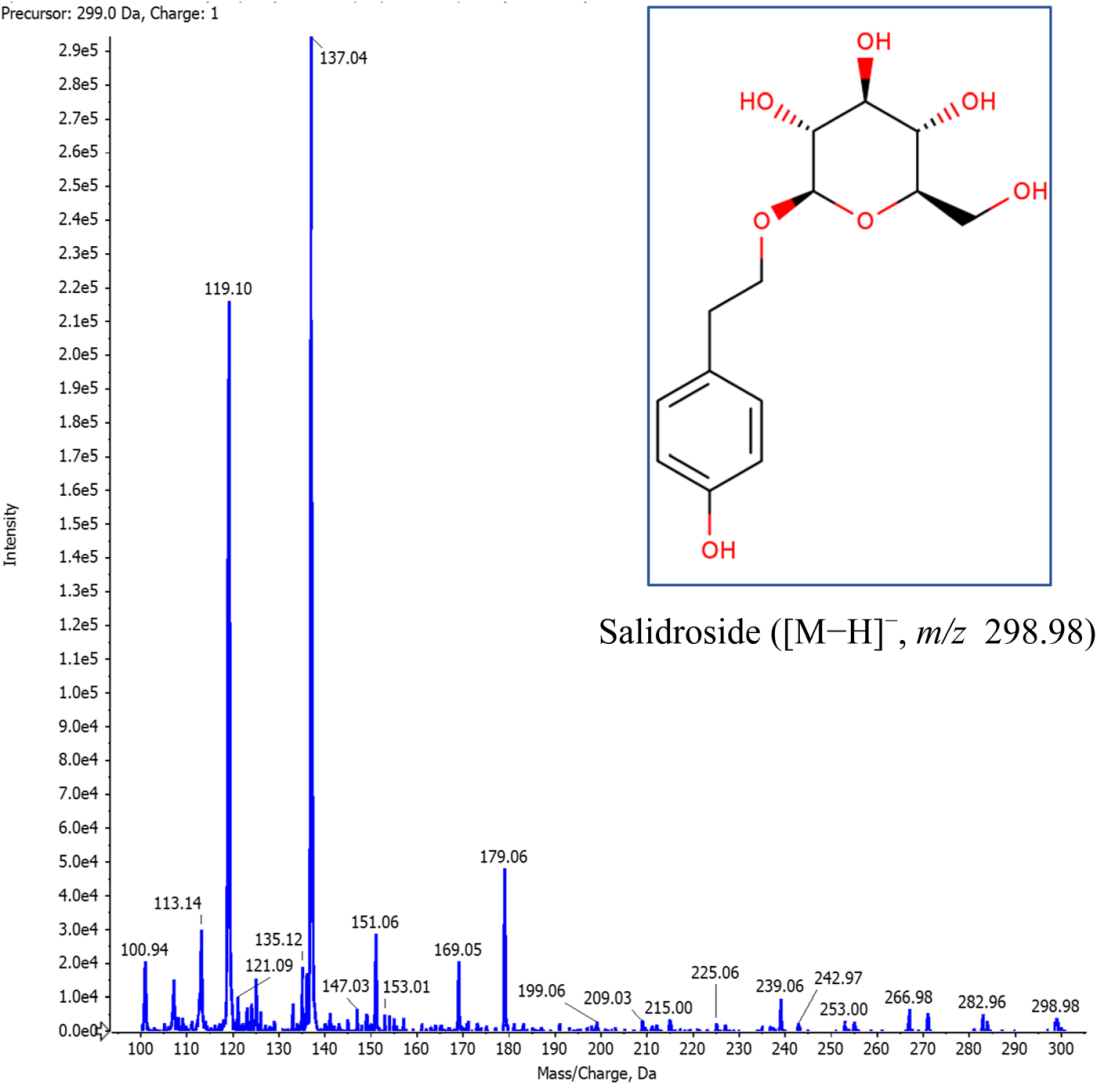
Mass spectrometric fragmentation pattern of deprotonated salidroside.

The final yield of RRME extraction was 5.6%. Eleven active compounds were detected in RRME (Table 1). Three compounds including salidroside (Fig. 2), rosavin and rosarin distinguish *Rhodiola rosea* from other species. Other compounds including *trans*-cinnamic alcohol derivatives (cinnamic acid, *trans*-cinnamic alcohol), tyrosol derivatives (*p*-tyrosol), phenolic compounds (gallic acid) and flavonoids (rutin, quercetin, quercitrin, kaempferol) were detected. The LC-MS analysis of RRME (Fig. 2) was consistent with findings reported in previous studies.^15,16,18^ The LC-MS fragmentation of Salidroside is presented in Fig. 3. The fragmentation patterns of the other 10 compounds are presented in Table 1 and the SI file (S1–S10).

**Table 1:** LC-MS analysis of phytochemical compounds of RRME

Number	RT (min)	Precursor ion [M–H]^−^ (*m*/*z*)	MS/MS fragment ions (*m*/*z*)	Identified compound	Reported biological activity	Ref.
**Tyrosol derivatives**						
1	2.47	298.98	113.14, 119.10, 179.06	Salidroside	Anti-inflammatory, neuroprotective, anticancer, antioxidant	15 and 16
2	3.62	137.00	119.03, 108, 104.94	*p*-Tyrosol	Cardioprotective, antioxidant	15

**Trans-cinnamic alcohol derivatives**						
3	9.21	426.97	233.03, 125.03, 133.06	Rosarin	Anti-inflammatory, neuroprotective, antioxidant	16
4	9.43	426.98	293.02, 149.00, 125.03	Rosavin	Antitumor, antioxidant	16
5	24.25	132.84	115.90, 104.81	*Trans*-cinnamic alcohol	Antioxidant	37
6	4.08	147.02	118.97, 102.92, 105.10	Cinnamic acid	Antioxidant, anti-inflammatory, analgesic	18 and 37

**Flavonoids**						
7	10.41	608.87	303.23, 301.00, 211.19	Rutin	Anti-inflammatory, antioxidant, antithrombotic, hepatoprotective, antiviral	17
8	11.80	446.95	300.98, 403.13, 191.07	Quercitrin	Anticancer, antioxidant, antibacterial	38
9	11.16	300.99	273.03, 151.14, 123.12	Quercetin	Antioxidant, anticancer	39 and 40
10	11.49	284.94	151.09, 133.08, 121.05	Kaempferol	Antitumor	40

**Phenolic compounds**						
11	2.72	169.05	124.98, 108.07	Gallic acid	Antifungal, antihyperglycemic, analgesic, antioxidant	18

### 4.3 Particle size and PDI determination

Significant changes in particle size and PDI were observed between the RRME-ICs and HPβCD. HPβCD displayed an average particle size of 516.4 nm and a heterogeneous system, as indicated by a PDI value of 0.375 ± 0.012. On the other hand, the RRME-ICs suspension showed improved stability, reduced particle size, and a less PDI, with an average size of 231.4 ± 0.45 nm and a PDI of 0.283 ± 0.01 as aligned with previous studies.^19,20^ These findings may be attributed to the inherent tendency of HPβCD to exhibit aggregation induced cyclodextrin's self-assembling nature in aqueous environments. Notably, the hydrophilic assemblies generated through cyclodextrin interactions and their complexes play a crucial role in solubilizing lipophilic compounds through complexation and/or the formation of micelle-like structures.

### 4.4 Morphological analysis

**Fig. 4:**
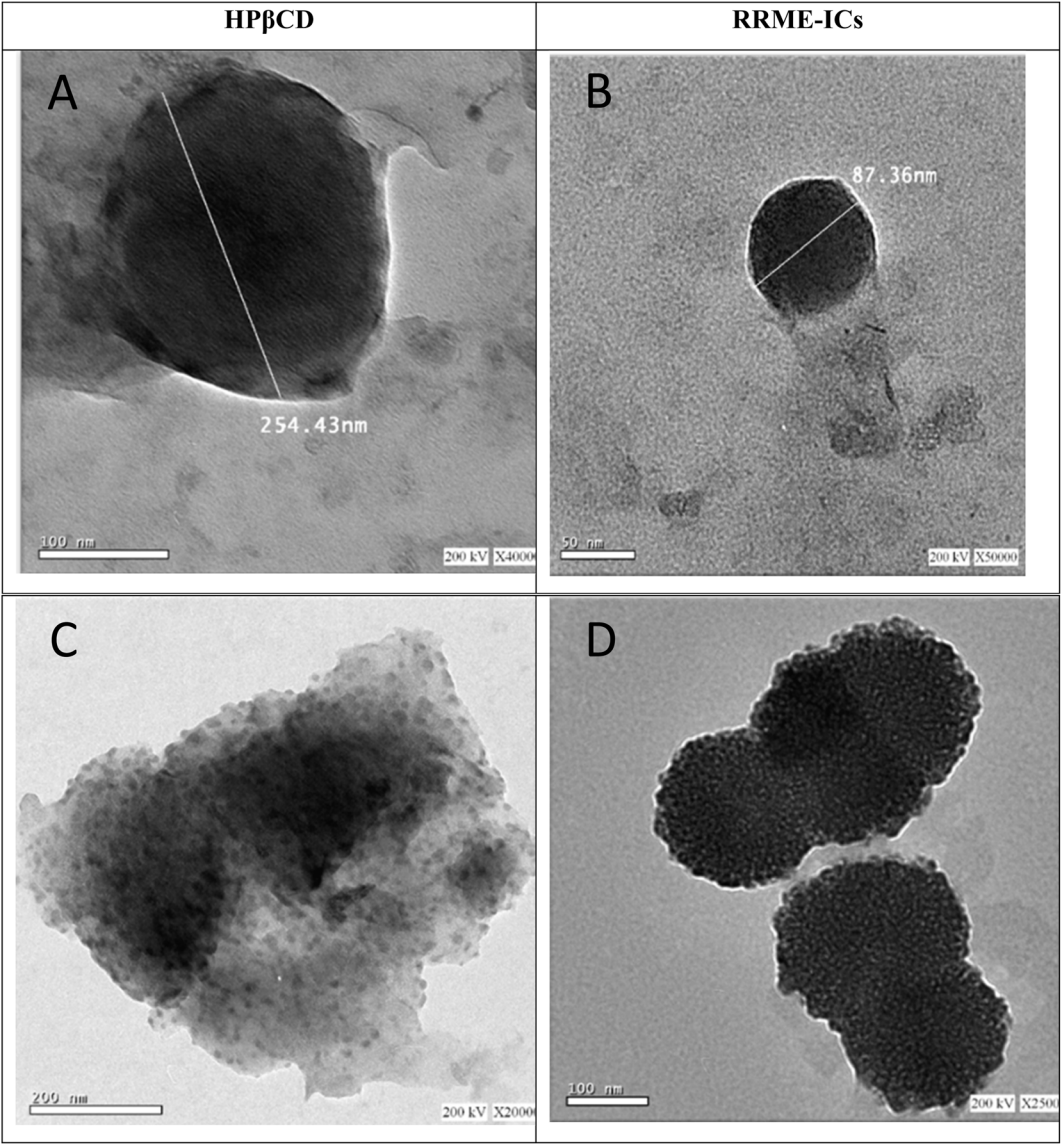
HR-TEM images of HPβCD and RRME-ICs. (A) HPβCD was found to form spherical vesicles that spontaneously self-assembled into larger structures, exhibiting an average particle size of 254.43 nm. (B) RRME-ICs exhibited nearly spherical vesicular structures, characterized by a thin encapsulating layer enveloping the RRME core, with an average particle size of 87.36 nm. (C) Agglomerated HPβCD exhibited enlarged vesicles with irregular shape, where larger particles appeared to attract and incorporate smaller ones, suggesting a heterogeneous self-assembly process. (D) Agglomerated RRME-ICs, similarly, displayed enlarged vesicular assemblies with irregular shape, where larger RRME-ICs particles appeared to attract and integrate smaller ones, indicative of a dynamic and size-dependent aggregation behavior.

HR-TEM images were obtained for HPβCD and RRME-ICs (Fig. 4). In the negative staining analysis, the particles appeared white against a dark background, with HPβCD particles showing empty interiors, distinct from RRME-ICs. The RRME-ICs predominantly appeared as spherical morphological vesicles with diameters range (87.36 to 166.5 nm) and were surrounded by a thin layer encasing the RRME. Additionally, some RRME-ICs displayed larger vesicles with irregular shapes. Evidence of particle agglomeration was also detected, with diameters with a range from 198.3 to 239.74 nm (Fig. 3), possibly indicating a tendency of larger particles to attract smaller ones.^24^ These results confirm the effective incorporation of RRME into its host HPβCD. In contrast, HPβCD presented round large vesicles. This procedure promotes cyclodextrin self-assembly, leading to a strong tendency for particle agglomeration in aqueous environments.^19^

### 4.5 FT-IR examination of HPβCD, RRME, and RRME-ICs

**Fig. 5:**
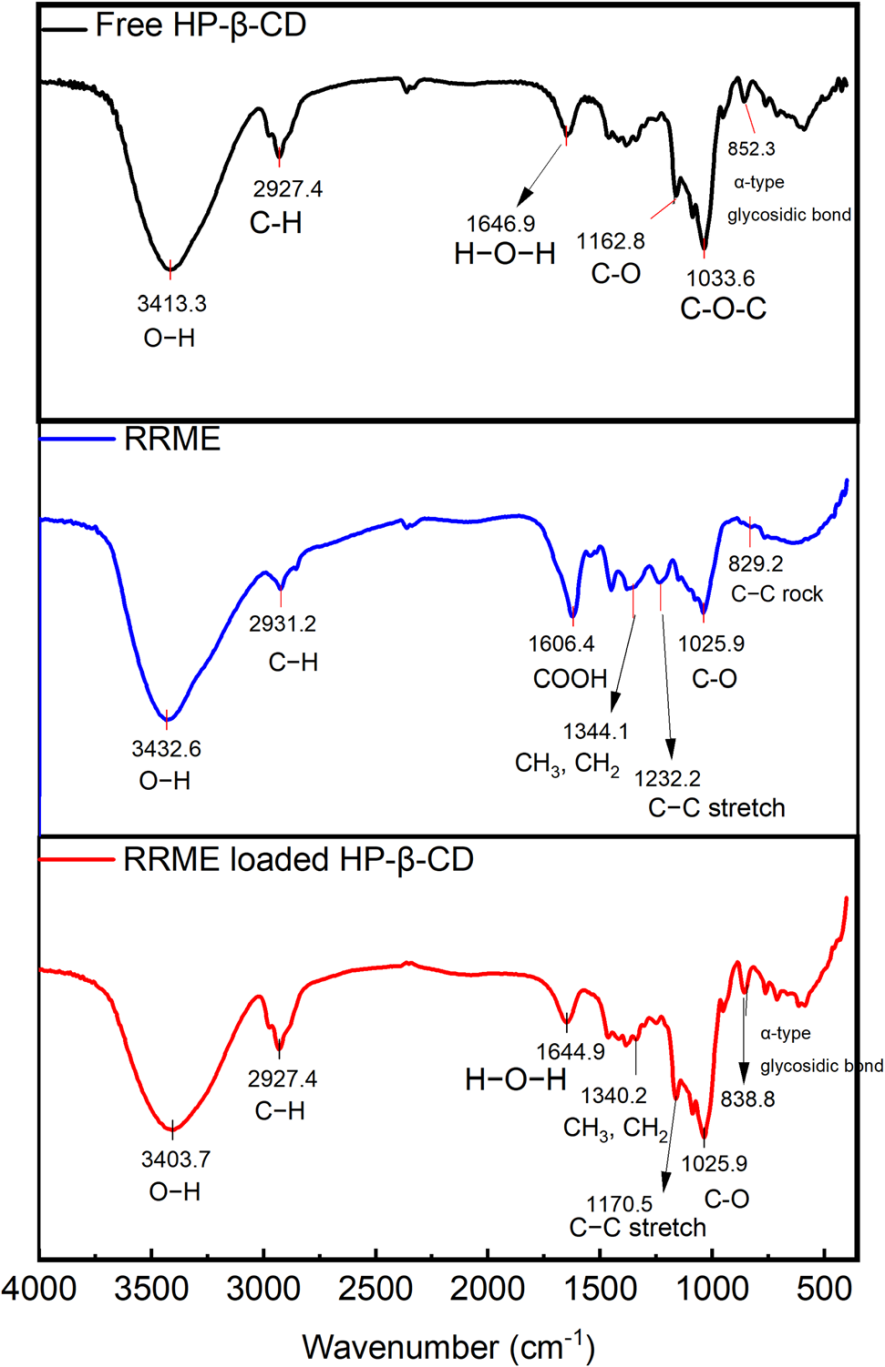
FT-IR spectra of RRME, RRME-ICs, and HPβCD.

The infrared spectral analysis of RRME demonstrated distinct vibrational features corresponding to key functional groups (Fig. 5). Peaks observed at approximately 3432 was referred to the O–H stretching vibration, while the band at 2931 cm^−1^ was indicative of the C–H anti-symmetric stretching vibration of methylene groups. Prominent peak at 1606 cm^−1^ was associated with carbonyl group of COOH functional groups. Additionally, band at 1344.1 cm^−1^ corresponded to the asymmetric deformation of CH_3_ and CH_2_ groups. The spectral range between 1232 cm^−1^ and 1025 cm^−1^ revealed vibrations associated with stretching of C–C and C–O, suggesting the detection of glycosides, carboxylic acids, and phenolic components.^22^ In comparison, the infrared spectra of HPβCD exhibited characteristic saccharide-associated bands, including 3413 cm^−1^ (O–H stretching vibration), 2927 cm^−1^ (C–H stretching vibration), 1646 cm^−1^ (O–H bending vibration) and 1162 cm^−1^ (C–O vibration). Notably, a distinct absorption peak at 852 cm^−1^ corresponded to the α-type glycosidic bond,^11^ indicative of the formation of glucopyranose units through α-1,4-glycosidic linkages.^11^ The absorption bands of RRME were largely obscured by the dominant spectral features of HPβCD such as the glycosidic bond signal. Also, certain shifts in band positions and reduced broadening of the O–H band were observed. This modification likely signifies the formation of hydrogen bonding between HPβCD and specific RRME components. Such observations possibly support the encapsulation of RRME constituents within the HPβCD cavity, as well as indicate potential external interactions. Moreover, the shift of the glycosidic bond signal to 838 cm^−1^ may represent the successful incorporation of RRME's hydrophobic components into HPβCD. Collectively, these findings support the development of stable ICs, as reported in previous studies.^19^

### 4.6 Entrapment efficiency (% EE) and drug loading (% DL) capacity

The RRME-ICs demonstrated remarkable entrapment efficiency of 78.88 ± 1.55% which could be due to the process of forming inclusion complexes. This process involved maintaining the complex solutions under well-protected and tightly sealed conditions throughout the preparation and drying stages for 3 days. Previous studies highlighted the impact of complexation duration and drying conditions on entrapment percentage of other natural extracts within HPβCD.^19,20,24,41^ These findings are further corroborated through the FT-IR results of RRME-ICs as discussed earlier. In addition, the %DL was 7.89 ± 1.45% for RRME-ICs. These results are consistent with findings of prior studies.^19,20,41^

### 4.7 ^1^H and 2D-NOESY NMR analysis

**Fig. 6:**
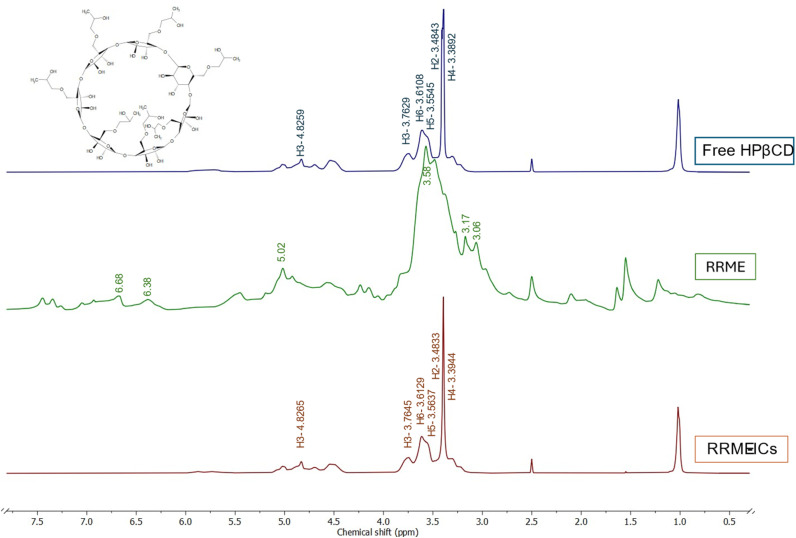
^1^H NMR spectra of free HPβCD, RRME, and RRME-ICs.

The ^1^H NMR spectra of RRME, HPβCD, and RRME-ICs are shown in Fig. 6. In the RRME spectrum, identification of peaks in the carbohydrate region (*δ* 3–5 ppm) of the ^1^H-NMR spectrum proved challenging due to the high glycoside content (including polysaccharides) and the presence of excipients in commercial *Rhodiola* products. Multiple resonances are detected corresponding to olefinic protons (*δ* 5.0–5.7 ppm) and methyl groups (*δ* 0.5–3 ppm) characteristic of terpenoids. Nevertheless, characteristic signals for salidroside and rosavin were discernible in the aromatic region (*δ* 6.2–8.5 ppm).^42,43^ Accordingly, these observations are consistent with and further corroborate the analytical results obtained from the FT-IR and LC-MS analyses. Furthermore, the ^1^H NMR spectrum of the RRME–ICs (Fig. 6) displayed proton signals attributable to both RRME and HPβCD.

Fig. 6 and Table 2 demonstrate the chemical shift variations of protons associated with the d-glucopyranose units of HPβCD. In the HPβCD molecule, H1 is located toward the middle of the cavity, H2 and H4 are positioned on the outer surface, H6 is situated at the outermost side, whereas H3 lies near the wider rim and H5 toward the narrower (bottom) side of the cavity. In the RRME–ICs spectrum, H1 and H2 exhibited negligible changes, while H3 and H6 showed slight downfield shifts and H4 and H5 displayed pronounced downfield shifts. This pattern is indicative of the successful inclusion of the aromatic and olefinic moieties of RRME within the hydrophobic cavity of HPβCD. The marked downfield displacement of H4 and H5 can be attributed to increased electron cloud density and the associated deshielding effects induced by the aromatic rings and double bonds of RRME, thereby supporting the effective formation of the RRME–HPβCD inclusion complexes.^19,20^

**Table 2:** Chemical shifts (*δ*) for HPβCD and RRME-ICs and differences in chemical shift (Δ*δ*)

Proton	Free HPβCD (*δ*/ppm)	RRME-ICs (*δ*/ppm)	Δ*δ* RRME-ICs/free HPβCD (Δ*δ*/ppm)
H1	4.8259	4.8265	−0.0006
H2	3.4843	3.4833	0.0010
H3	3.7629	3.7645	−0.0016
H4	3.3892	3.3944	−0.0052
H5	3.5545	3.5637	−0.0092
H6	3.6108	3.6129	−0.0021

**Fig. 7:**
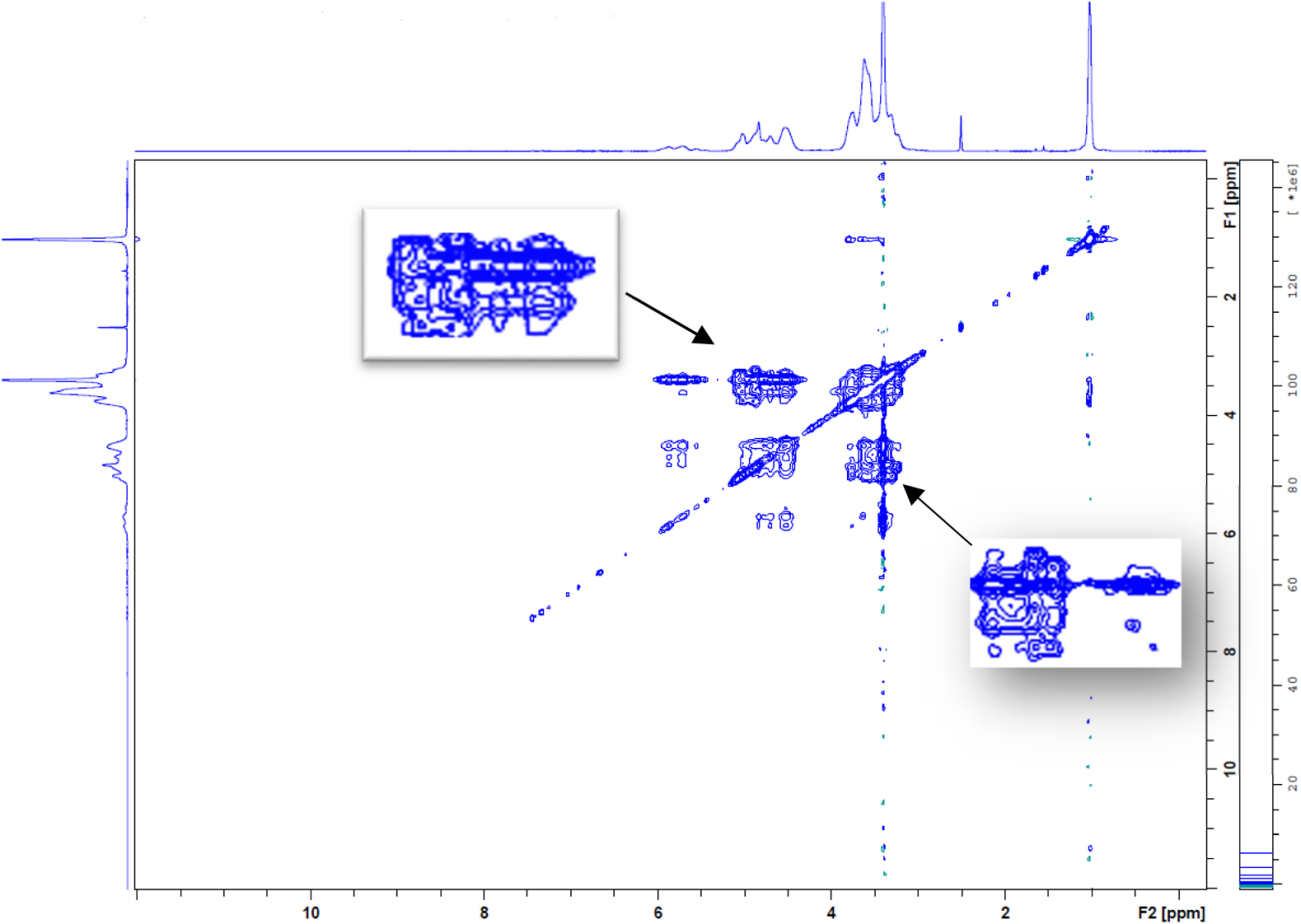
2D-NOESY spectrum of RRME-ICs demonstrated distinct cross-peaks, indicative of spatial proximity between RRME protons and HPβCD inner cavity, confirming successful inclusion complex formation. Notably, the chemical environment surrounding the H3 and H5 protons of the inner cavity of HPβCD was altered upon complexation, leading to deshielding effects observable in the NMR spectrum.

To gain deeper insight into the encapsulation of RRME within the cavity of HPβCD, 2D nuclear Overhauser effect spectroscopy (NOESY) was executed on the RRME-ICs (Fig. 7). This method facilitates the detection of cross-peaks suggestive of spatial proximity between HPβCD and RRME. NOE provides insight into spin polarization transfer between two distinct populations in close spatial proximity, specifically within a distance of 0.4 nm. In this study, 2D-NOESY of RRME-ICs revealed pronounced cross peaks between H3 and H5 protons of HPβCD and the protons of RRME constituents. These correlations confirm the spatial proximity required for NOE interactions and are consistent with previously reported chemical shifts.^19,20,41^ Collectively, these results strongly support the encapsulation of RRME phytochemicals within the HPβCD. This observation aligns with prior studies demonstrating the effective inclusion of diverse guest molecules into HPβCD using 2D-NMR techniques.

### 4.8 Thermogravimetric analysis

**Fig. 8:**
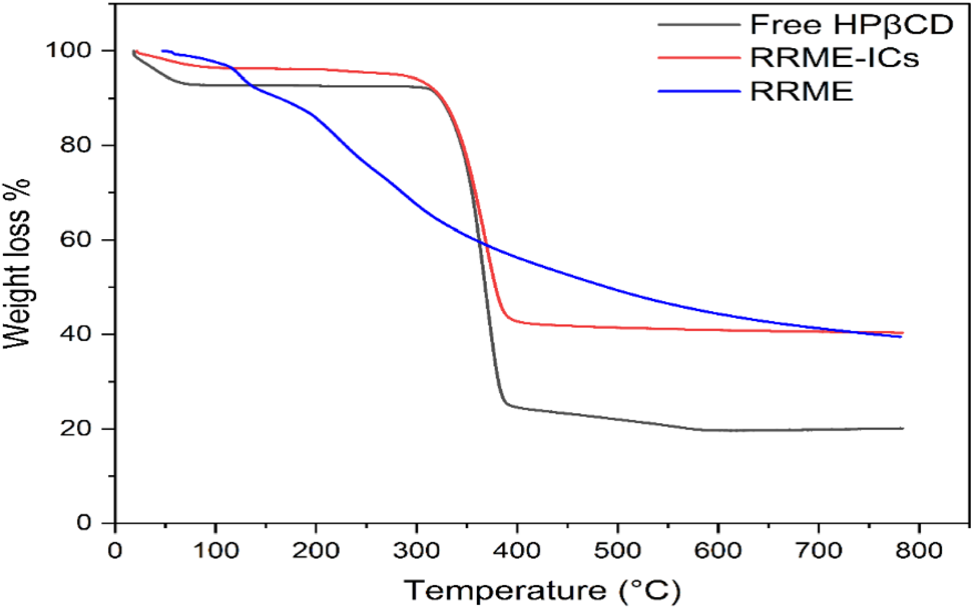
TGA graph of RRME, RRME-ICs, and HPβCD.

Thermogravimetric analysis (TGA) of the pure extract exhibited a gradual mass loss with increasing temperature, indicative of the thermal degradation of extract components (Fig. 8). Weight loss of RRME was gradually observed with surging the temperature, corresponding to the decomposition of thermolabile constituents. Despite this degradation, the extract retained a portion of its mass at higher temperatures, suggesting the presence of components with inherent thermal resistance.^44^ Conversely, the RRME-loaded HPβCD inclusion complex demonstrated a markedly improved thermal stability profile. The thermogram (Fig. 8) revealed a delayed onset of decomposition compared to the pure extract, with substantial weight loss occurring at higher temperatures around 350 °C. The total mass loss over the temperature range was less pronounced for the inclusion complex. This enhanced thermal stability is evidenced to the encapsulation of RRME within the hydrophobic cavity of HPβCD, which possibly created a protective microenvironment. The host–guest interactions between HPβCD and RRME are likely responsible for mitigating the volatilization and decomposition of extract components by shielding them from external thermal energy.^11,41^ These findings support the encapsulation of RRME into HPβCD significantly enhancing its thermal stability, rendering the inclusion complex more suitable for applications involving elevated temperatures. The improved thermal behavior of the RRME-loaded HPβCD inclusion complex has potential advantages in pharmaceutical formulations, food preservation, and industrial processes where thermal degradation of bioactive compounds presents a challenge.

### 4.9 Drug release profile

**Fig. 9:**
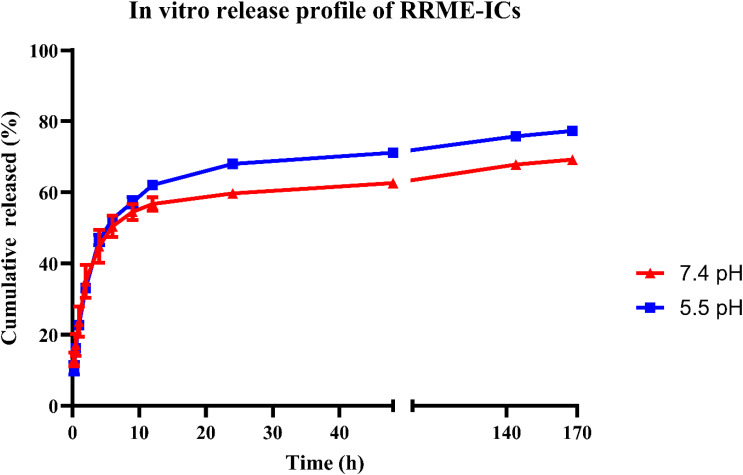
*In vitro* release profile of RRME-ICs in PBS (pH 7.4 and pH 5.5) at 37 °C (*n* = 3).

*In vitro* release kinetics of RRME-ICs were assessed under two distinct pH conditions: physiological (pH 7.4) and acidic (pH 5.5). Under pH 7.4, the formulation exhibited a sustained release pattern, with cumulative release reaching approximately 59% within the first 24 h. While exposure to pH 5.5 resulted in a comparatively accelerated release, achieving 68% over the same time interval (Fig. 9).

### 4.10 Release kinetics modeling

The drug release profile of RRME-ICs was analyzed using multiple kinetic models, including zero-order, first-order, Higuchi, and Korsmeyer–Peppas, as presented in the SI file (S11, S12) and Table 3. The coefficient of determination (*R*^2^) values were calculated to identify the model that best describes the release kinetics.^26^

**Table 3:** Correlation coefficients (*R*^2^) of drug release kinetics models (zero-order, first-order, Higuchi and Korsmeyer–Peppas model) applied to RRME-ICs at pH 7.4 and pH 5.5

Model	*R* ^2^ (pH 7.4)	*R* ^2^ (pH 5.5)
Zero-order	0.4279	0.4448
First-order	0.5516	0.5999
Higuchi	0.6329	0.6568
Korsmeyer–Peppas	0.8531	0.8591

The release behavior of RRME-ICs was best described by the Korsmeyer–Peppas model, as evidenced by the highest *R*^2^ values at both pH 5.5 and 7.4. This model is suited for systems where the release mechanism follows Fickian diffusion.^45^

The Korsmeyer–Peppas release exponent (*n*) was calculated to be 0.29, which is below the threshold of 0.5. This indicates that the release mechanism is governed by Fickian diffusion, where the plant extract diffuses through the hydrated HPβCD matrix primarily under the influence of a concentration gradient.^27^ The low *n* value suggests minimal contribution from matrix relaxation or erosion, highlighting diffusion as the dominant release pathway. The preferential release observed under acidic conditions (pH 5.5) further supports the potential of HPβCD complexes for targeted delivery in tumor microenvironments, where acidic pH enhances diffusion-driven release. These findings align with previous studies demonstrating the utility of cyclodextrins to enhance solubility and control release of plant-based extracts.^46,47^

### 4.11 Assay of total phenolic content

Standard calibration curve was conducted using gallic acid against the Folin–Ciocalteu reagent. The calculated linear regression equation was *y* = 3.0811*x* + 0.17, and the calibration data are provided in the SI file (S13). The elevated antioxidant activity observed in the RRME can be attributed to its substantial phenolic content. Specifically, 1 mg of the RRME was found to be equivalent to 249 µg of gallic acid which is determined by the total phenolic compounds in the RRME. A similar correlation between phenolic content and antioxidant activity was reported which could be attributed to presence of gallic acid, quercitrin, quercetin, kaempferol, and rutin which have antioxidant potential.^28^

### 4.12 Radical scavenging activity assay

DPPH (2,2-diphenyl-1-picrylhydrazyl) is a thermally stable radical capable of receiving either an electron or a hydrogen atom, resulting in the generation of a diamagnetic, stable molecule. The reduction potential of DPPH radicals was assessed by monitoring the decrease in absorbance at 517 nm, which is induced by the presence of antioxidants^48^ as provided in the SI file (S14 and S15). The DPPH radical scavenging capacity of RRME revealed a concentration-dependent effect within the range of 31.25–500 µg mL^−1^. RRME and ascorbic acid (500 µg mL^−1^ each) achieved inhibition rates of 92.06% and 95.56%, respectively. The IC_50_ values were measured to be 64.7 µg mL^−1^ for RRME and 14.0 µg mL^−1^ for ascorbic acid (Table 4). These findings align with previous studies.^5,28^

**Table 4:** IC_50_ values and RSD% for ascorbic acid, RRME and RRME-ICs

	IC_50_ (µg mL^−1^)	RSD%
Ascorbic acid	14.0	95.56
RRME	64.7	92.02
RRME-ICs	65.7	92.60

### 4.13 Antimicrobial activity

The antimicrobial actions of RRME and RRME-ICs were evaluated using *Pseudomonas putida*, *Escherichia coli*, and *Staphylococcus aureus*. Table 5 presents the minimum inhibitory concentration (MIC90) values for RRME, and RRME-ICs against the three bacterial strains. The antimicrobial properties of RRME are attributed to its rich contents of *trans*-cinnamic alcohol derivatives (rosavin, rosarin, rosin) and tyrosol derivatives such as salidroside.^3,5,49^ These components were reported to increase bacterial membrane permeability.^50^ HPβCD exhibited no antimicrobial activity against the tested bacterial strains as reported previously.^20^ The MIC90 of RRME exceeded 20 mg mL^−1^ against the three bacterial strains, indicating limited antibacterial potency. On the other hand, RRME-ICs exhibited significantly enhanced activity, with IC_90_ values of 2.38, 3.95, and 1.92 mg mL^−1^ against *E. coli*, *P. putida*, and *S. aureus*, respectively. This represents over 10-fold improvement compared to the extract as shown in Table 5. These findings highlight the significance of encapsulating RRME within HPβCD to enhance its antimicrobial efficacy.^19–21^

**Table 5:** IC_90_ values for RRME and RRME-ICs against *E. coli*, *P. putida*, and *S. aureus*

IC_90_ (mg mL^−1^)		
Bacterial strain	RRME	RRME-ICs
*Escherichia coli*	>20	2.38
*Pseudomonas putida*	>20	3.95
*Staphylococcus aureus*	>20	1.92

### 4.14 Anti-melanoma activity assay

**Fig. 10:**
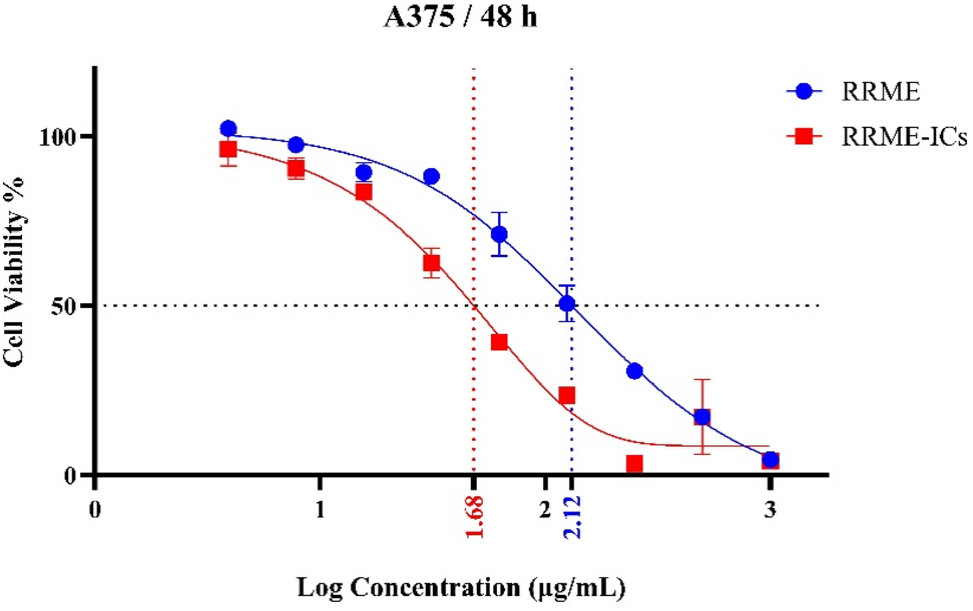
Evaluation of RRME and RRME-ICs cytotoxicity on A375 cell line at variable concentrations using Alamar Blue assay. Data are presented as mean (%) of control ± SE, *n* = 3. The IC_50_ of RRME and RRME-ICs were 131.24 and 48.14 µg mL^−1^, respectively.

Cell viability of Human malignant melanoma A375 cells treated with different concentrations (7.813–1000 µg mL^−1^) of RRME and RRME-ICs were evaluated using Alamar blue assay (Fig. 10). After 48 h of incubation, A375 cell line showed a significant reduction (*p* < 0.05) in cell viability upon treatment with the RRME-ICs, with IC_50_ value of 48.14 µg mL^−1^, compared to cells treated with similar concentrations of RRME, with IC_50_ value of 131.24 µg mL^−1^ (Fig. 10). The anticancer potential of RRME and RRME-ICs is possibly due to the presence of salidroside, a phenylethanoid glycoside which is considered the primary anticancer agent. Other molecules such as rosavin, rosarin, rosin (glycosides with adaptogenic and cytotoxic effects) could have also contributed to the observed cytotoxicity. Finally, flavonoids, including kaempferol and quercetin, modulate signaling pathways can enhance the anticancer potential, through disturbing tumor cell structure and promoting DNA fragmentation, inhibition of proliferation and metastasis, impairing blood vessel formation in tumors, and reshaping tumor metabolism and reducing growth.^51–53^ It is of note that the HPβCD host displayed no cytotoxic effect. Evidently, RRME exhibited increased cytotoxicity upon complexation with HPβCD. Several studies recognized HPβCD as an effective host for improving the therapeutic efficacy of both natural and synthetic drugs with anticancer potential.^54–56^ In addition to enhancing solubility, stability, with preserving bioactivity, HPβCD serves to shield loaded compounds from degradation and promotes their transport toward the intended biological target.^57^

## 5 Conclusions

In this study, RRME was extracted using the Soxhlet extraction method, followed by a detailed chemical analysis conducted using LC-MS. Subsequently, RRME was entrapped into HPβCD, and the physicochemical properties of the resulting RRME-ICs elucidated. HR-TEM analysis revealed the formation of round vesicles (87.36 to 166.5 nm) along with larger agglomerates (198.3 to 239.74 nm) and a PDI of 0.283 ± 0.01. The entrapment efficiency (%EE) of RRME-ICs was 78.88%. ^1^H NMR, 2D NMR NOESY, HR-TEM, and FT-IR demonstrated the encapsulation of RRME into HPβCD. TGA results demonstrated an improvement in thermal stability profile upon encapsulation in HPβCD. Release kinetics analysis confirmed that the Korsmeyer–Peppas model provided the best fit for RRME-ICs at physiological and acidic pH conditions. Furthermore, RRME-ICs showed enhanced antibacterial efficacy against *Pseudomonas putida*, *Staphylococcus aureus*, and *Escherichia coli*. In addition, RRME-ICs demonstrated promising anticancer activity against human melanoma A375 cells. The encapsulation of RRME into HPβCD not only improved its stability profile but also increased its antimicrobial and anticancer activities while preserved its antioxidant activity. These findings support the future application of similar inclusion complexes in controlling microbial growth and contamination in food systems and advancing therapeutic interventions in cancer treatments.

## Conflicts of interest

There are no conflicts of interest to declare.

## Supplementary Material

RA-016-D5RA07949G-s001

## Data Availability

The authors confirm that the data supporting the findings of this study are available within the article and its supplementary information (SI). Raw data that support the findings of this study are available from the corresponding author, upon reasonable request. Supplementary information: mass spectra of bioactive constituents from the RRME, kinetic modeling of RRME-ICs, calibration curves for phenolic compounds obtained *via* the Folin–Ciocalteu method, and radical scavenging activity profiles. See DOI: https://doi.org/10.1039/d5ra07949g.
