# Supplementary material for: Nanoencapsulation of Rhodiola rosea extract into 2-hydroxypropyl-β-cyclodextrin: enhanced antibacterial and anticancer activities
Source: RSC Adv. 2026 Feb 26;16(12):11271–83. doi: 10.1039/d5ra07949g (PMC12937486; doi:10.1039/d5ra07949g)
Supplement: RA-016-D5RA07949G-s001 [file RA-016-D5RA07949G-s001.pdf]

Supplementary Information (SI) for RSC Advances.
This journal is © The Royal Society of Chemistry 2026

Nanoencapsulation of *Rhodiola rosea* extract into 2-Hydroxypropyl-β-Cyclodextrin: Enhanced Antibacterial and Anticancer Activities

Shaza Khorshed[1], Ahmed Maher Abdeldayem[1], Wolfgang Fritsche[2], Hassan Mohamed El-Said Azzazy[1,2]

[1]Department of Chemistry, School of Sciences & Engineering, The American University in Cairo, New Cairo, Egypt 11835

[2]Department of Nanobiophotonics, Leibniz Institute of Photonic Technology, Jena 07745, Germany

Corresponding author.

Prof. Hassan M. E. Azzazy

Email: hazzazy@aucegypt.edu

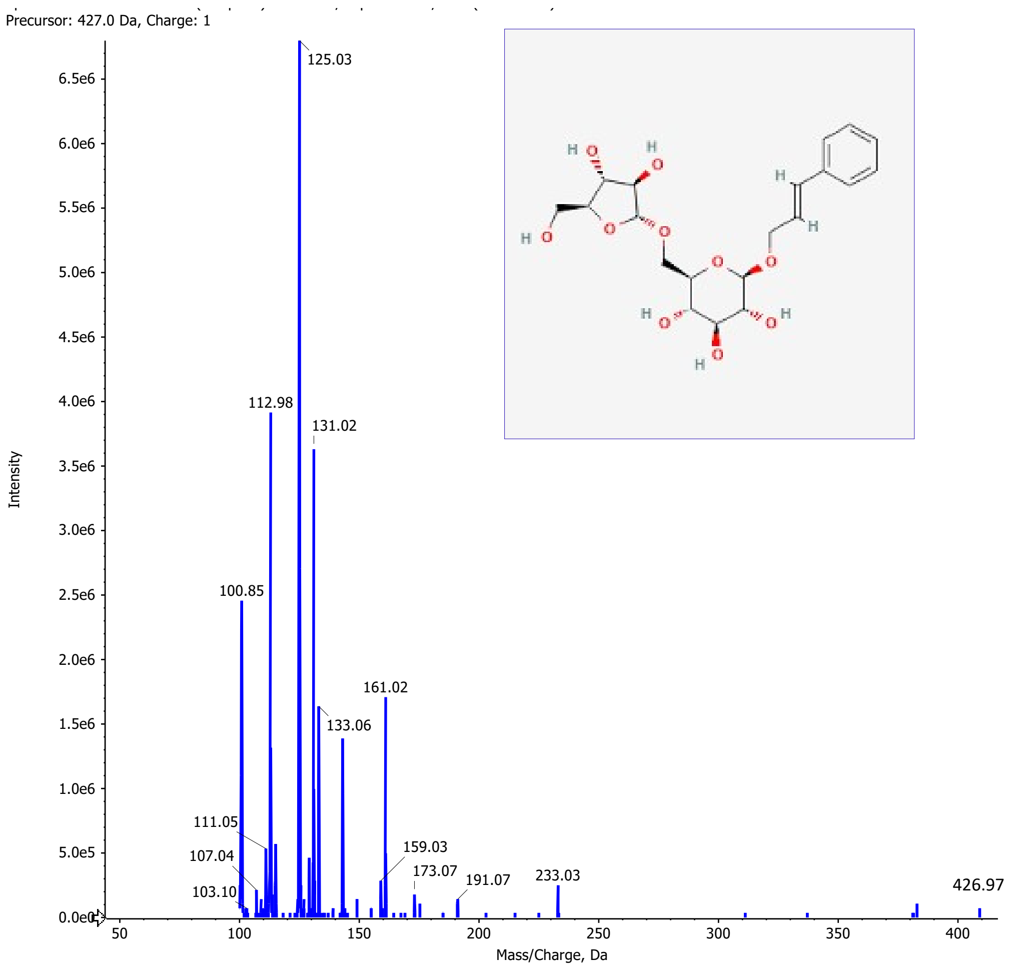

S1. Mass spectrum of Rosarin ([M−H]$^{-}$, *m/z* 426.97).

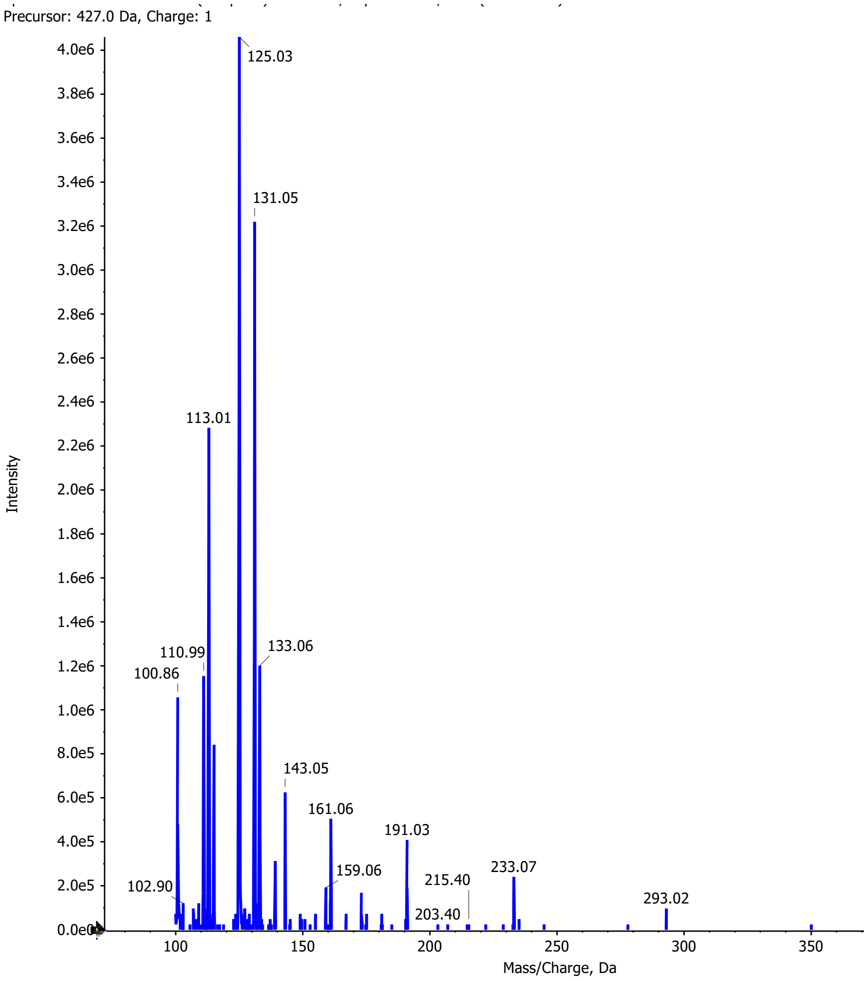

Precursor: 427.0 Da, Charge: 1
Intensity
Mass/Charge, Da
125.03
131.05
113.01
110.99
100.86
133.06
143.05
161.06
191.03
159.06
233.07
215.40
102.90
203.40
293.02

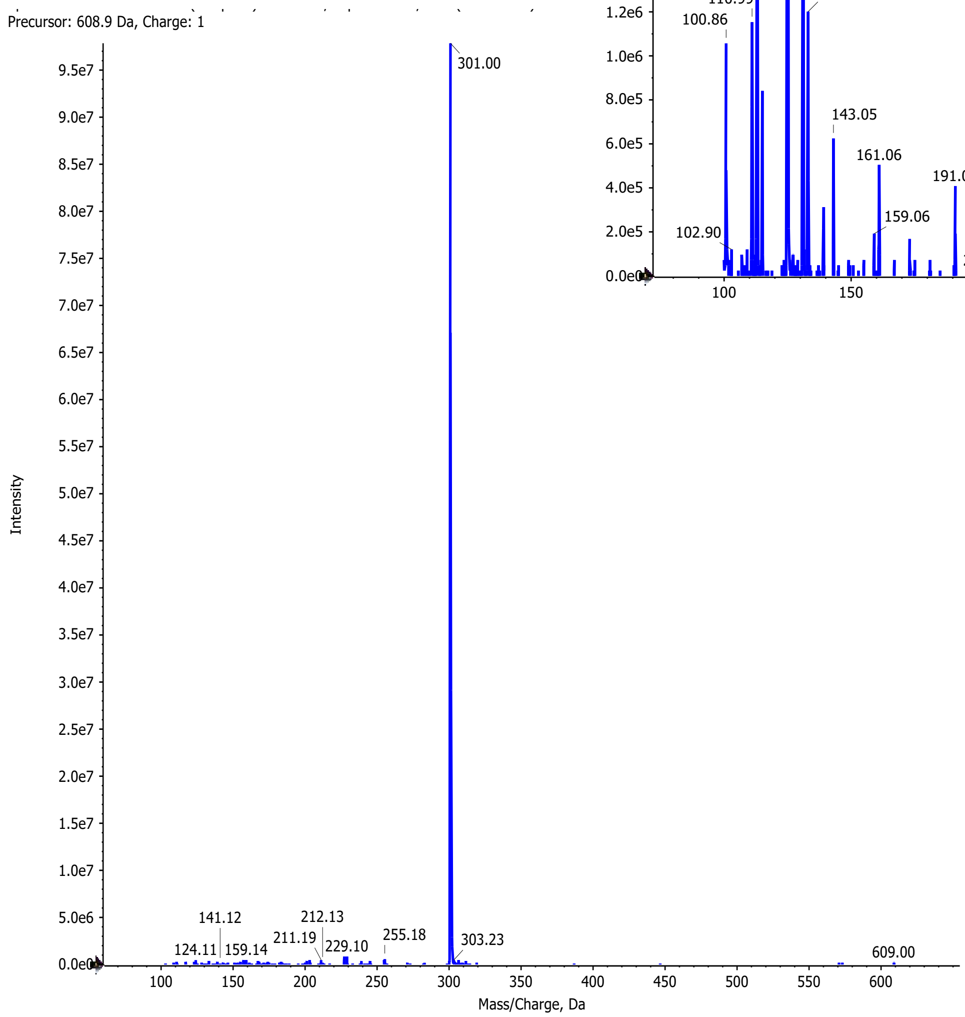

Precursor: 608.9 Da, Charge: 1
Intensity
Mass/Charge, Da
301.00
141.12
212.13
124.11
159.14
211.19
229.10
255.18
303.23
609.00

S2.

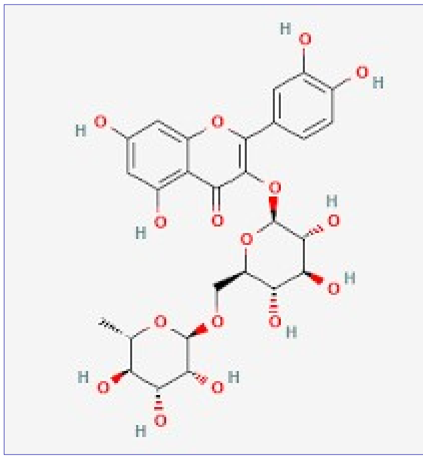

S3. Mass spectrum of Rutin ([M−H]$^-$, *m/z* 608.9).

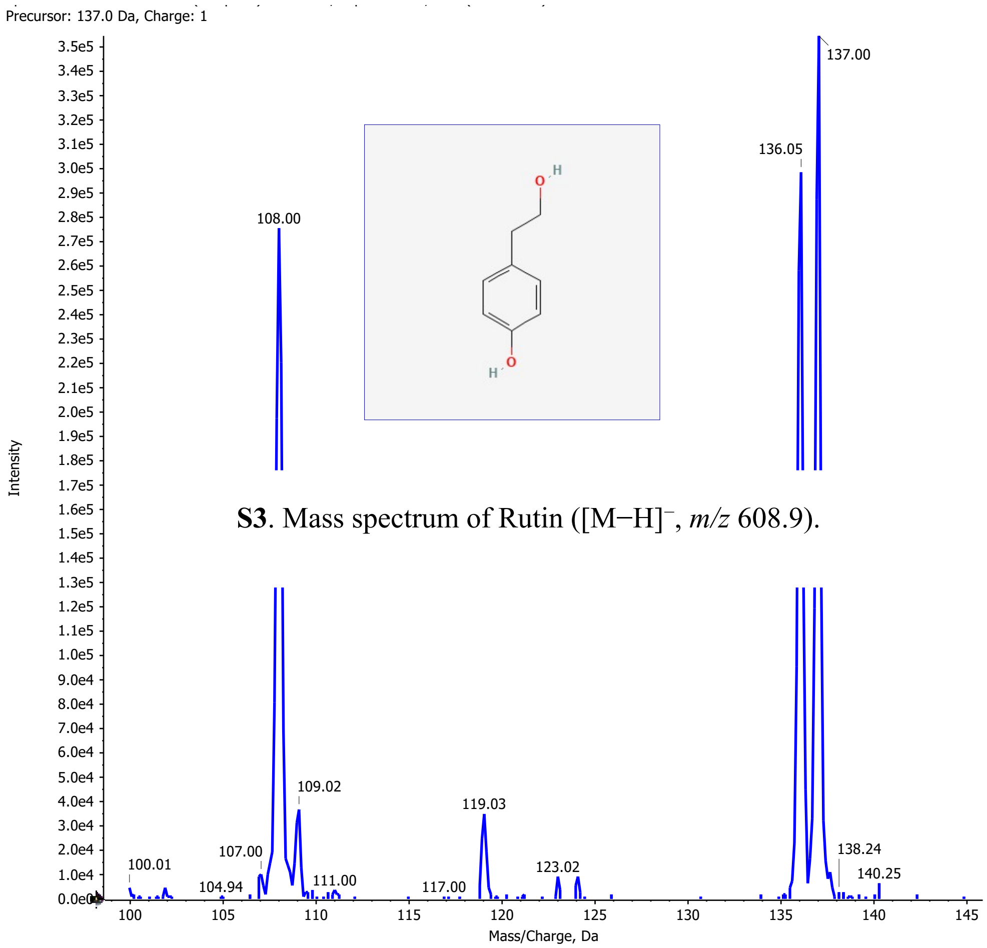

S4. Mass spectrum of *p*-Tyrosol ([M−H]$^-$, *m/z* 137).

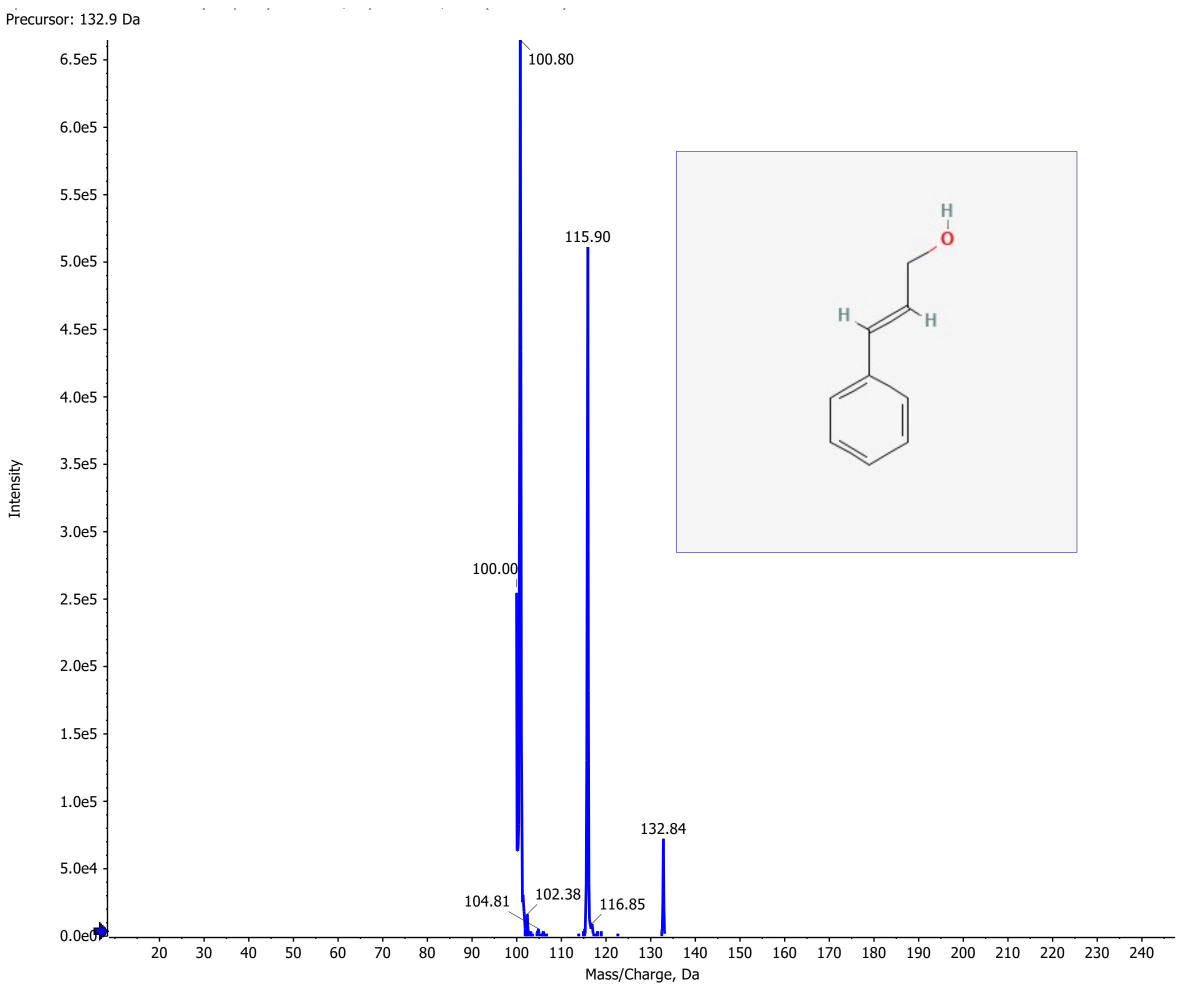

S5. Mass spectrum of Trans-cinnamic alcohol ([M−H]$^{-}$, *m/z* 132.84).

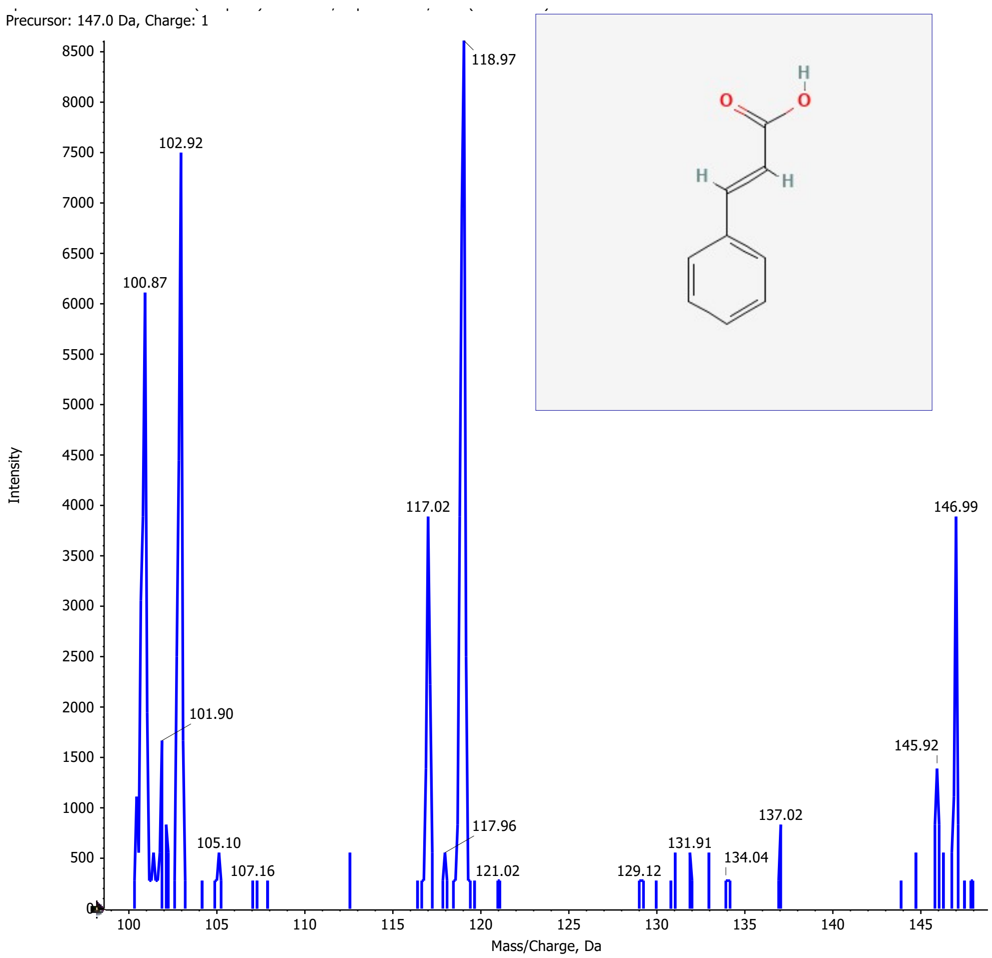

S6. Mass spectrum of Cinnamic acid ([M−H]$^-$, *m/z* 147.02).

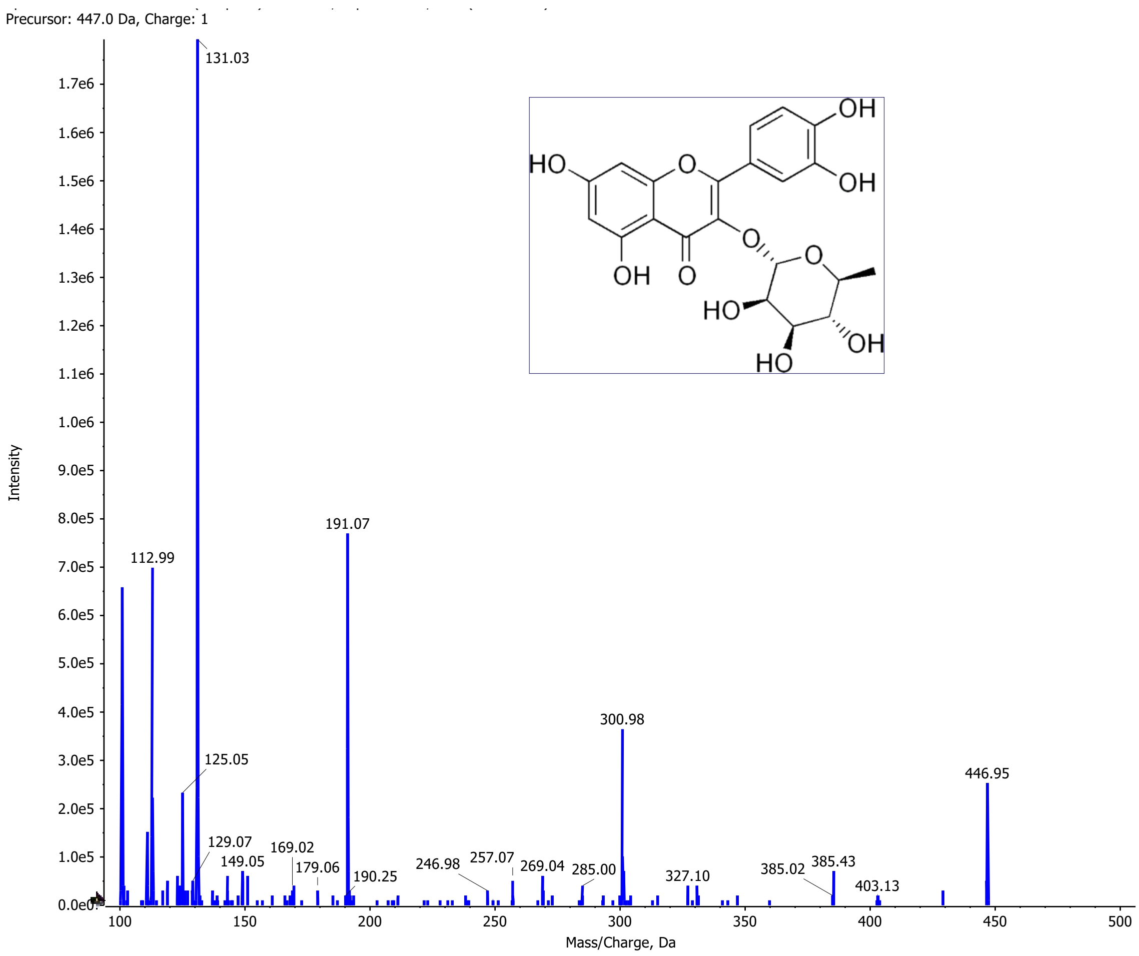

S7. Mass spectrum of Quercitrin ([M−H]$^{-}$, *m/z* 446.95).

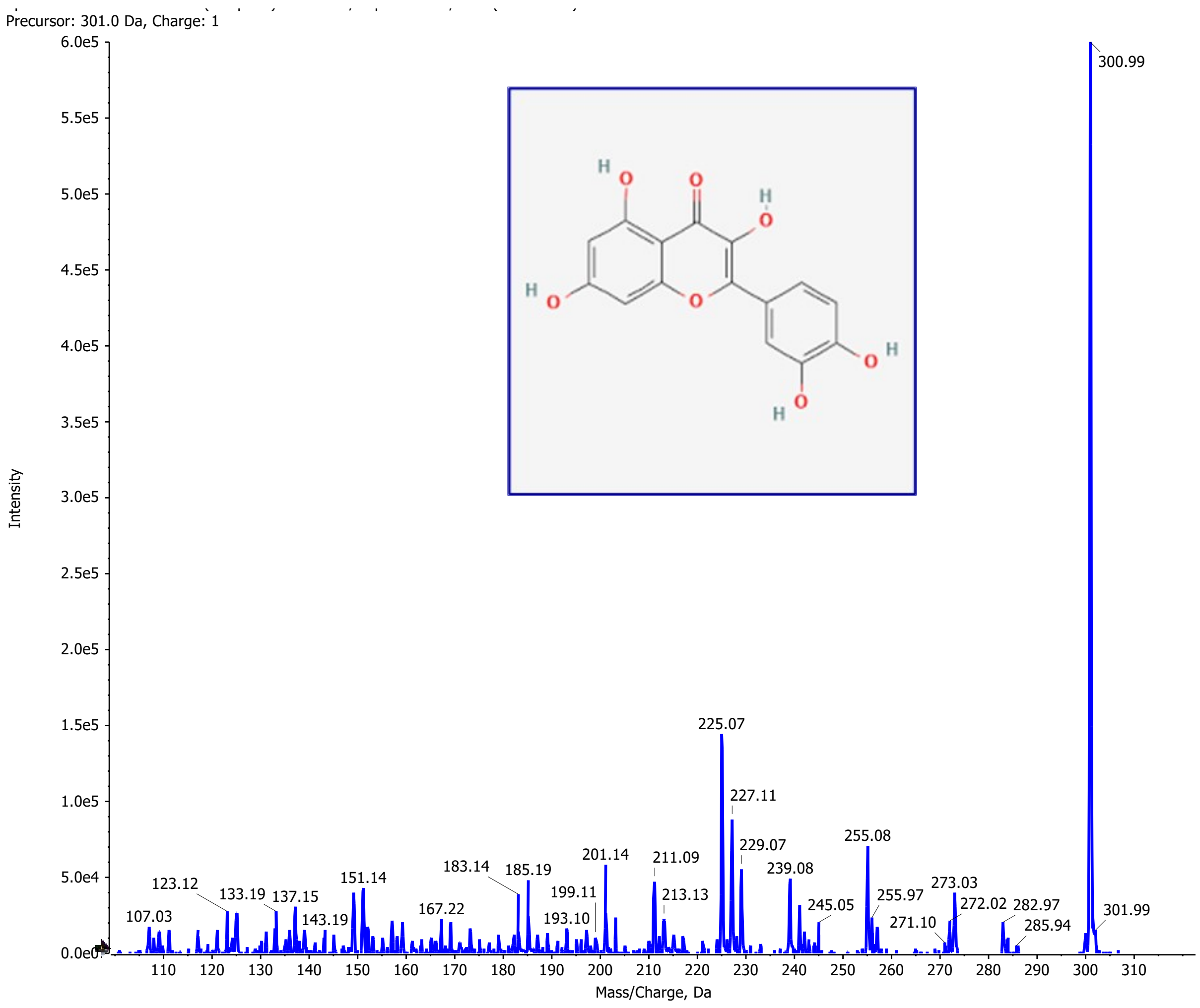

S8. Mass spectrum of Quercetin ([M−H]$^-$, *m/z* 300.99).

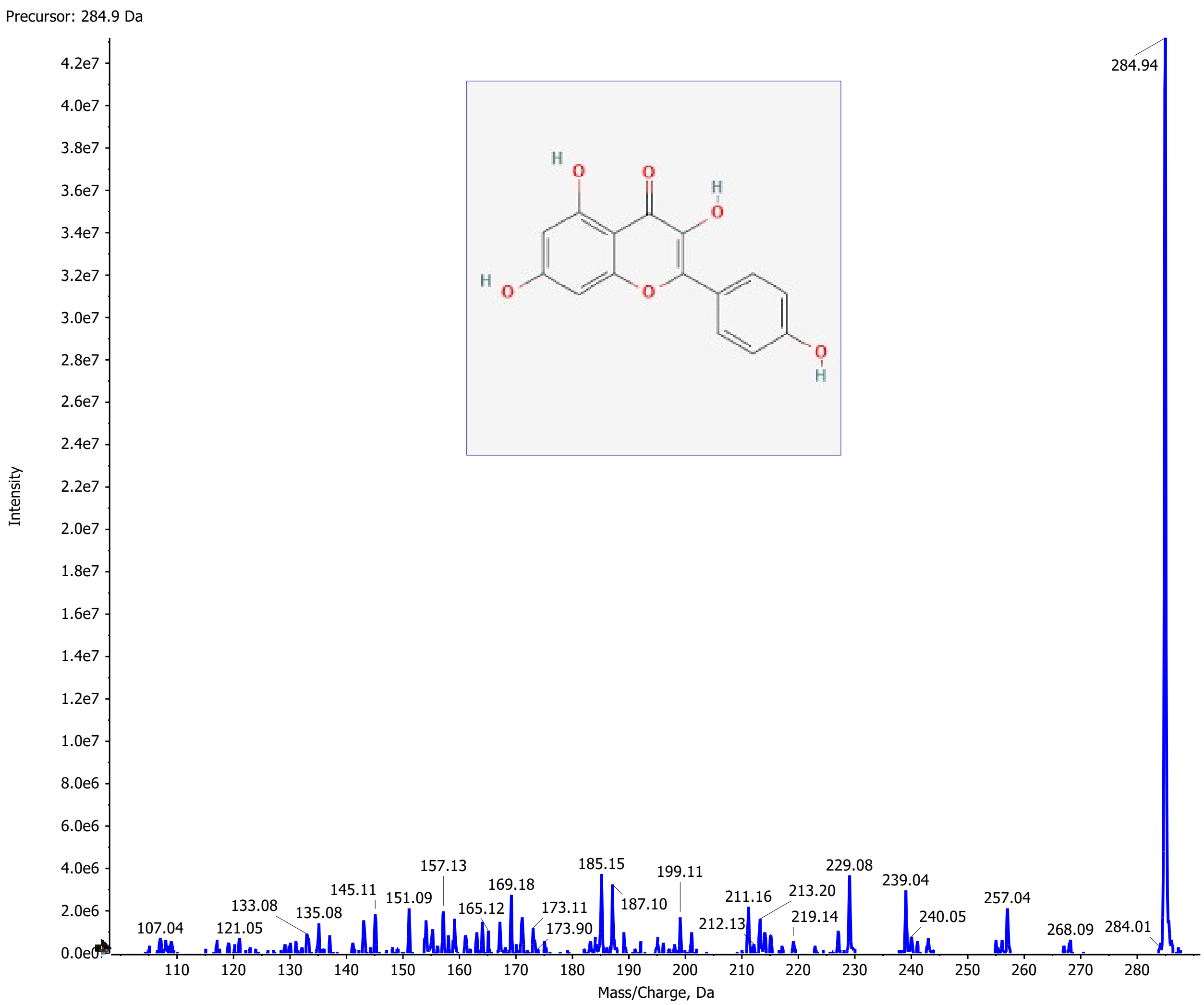

S9. Mass spectrum of Kaempferol ([M−H]$^{-}$, *m/z* 284.98).

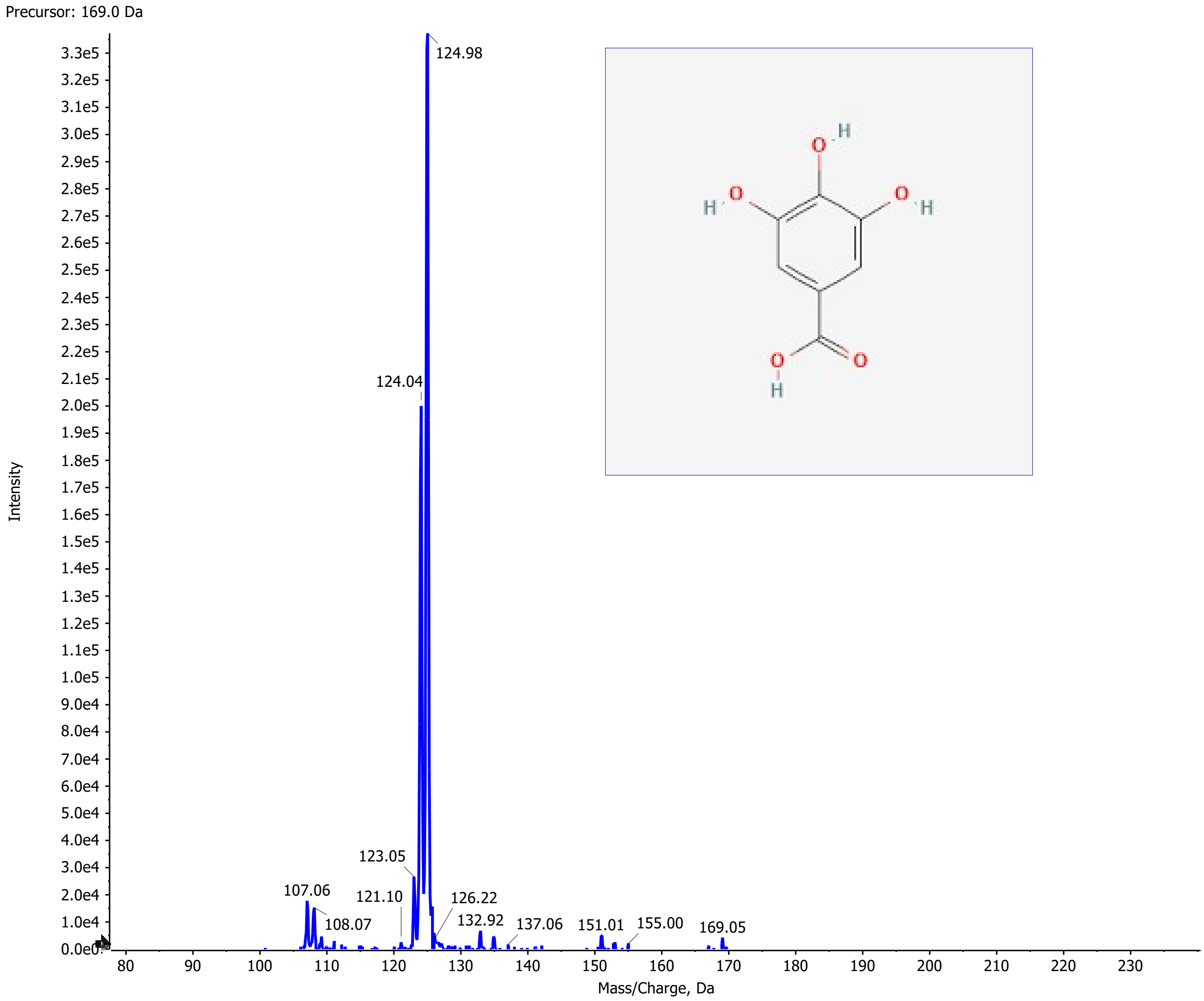

S10. Mass spectrum of Gallic acid ([M−H]$^-$, *m/z* 169.05).

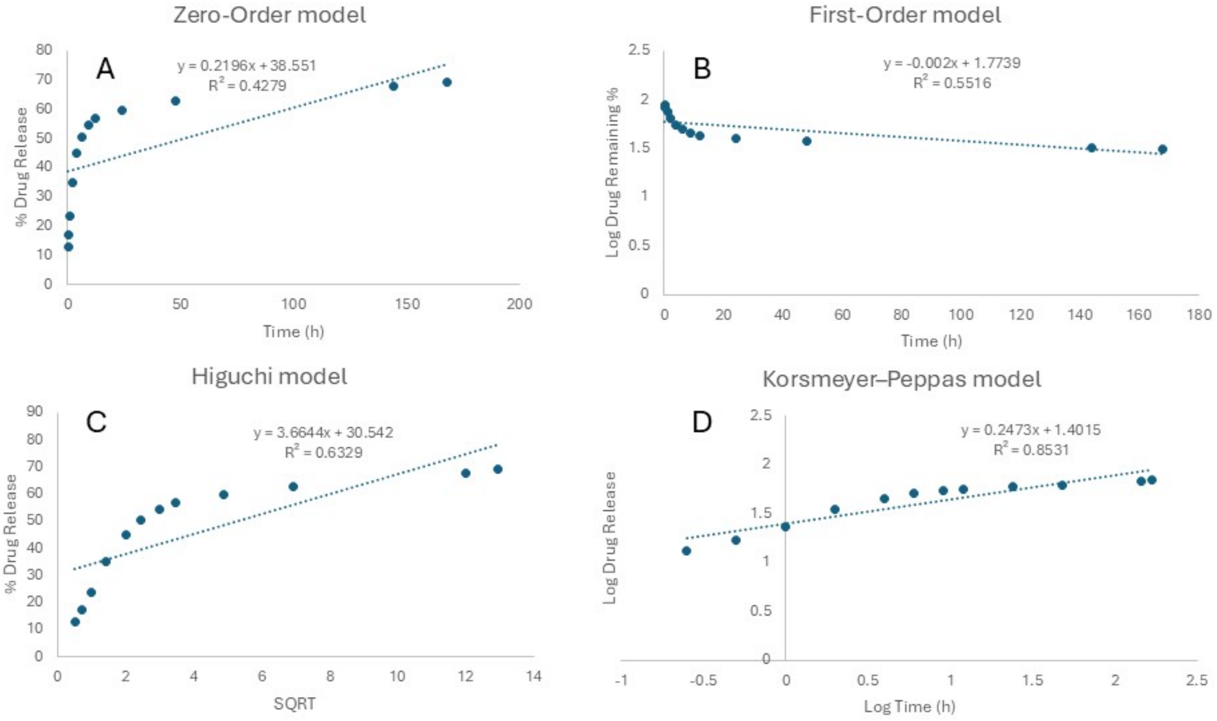

S11. Release kinetics model fitting curves of **RRME-ICs** at pH 7.4: (A) zero-order model; (B) first-order kinetics model; (C) Higuchi model; and (D) Korsmeyer–Peppas model.

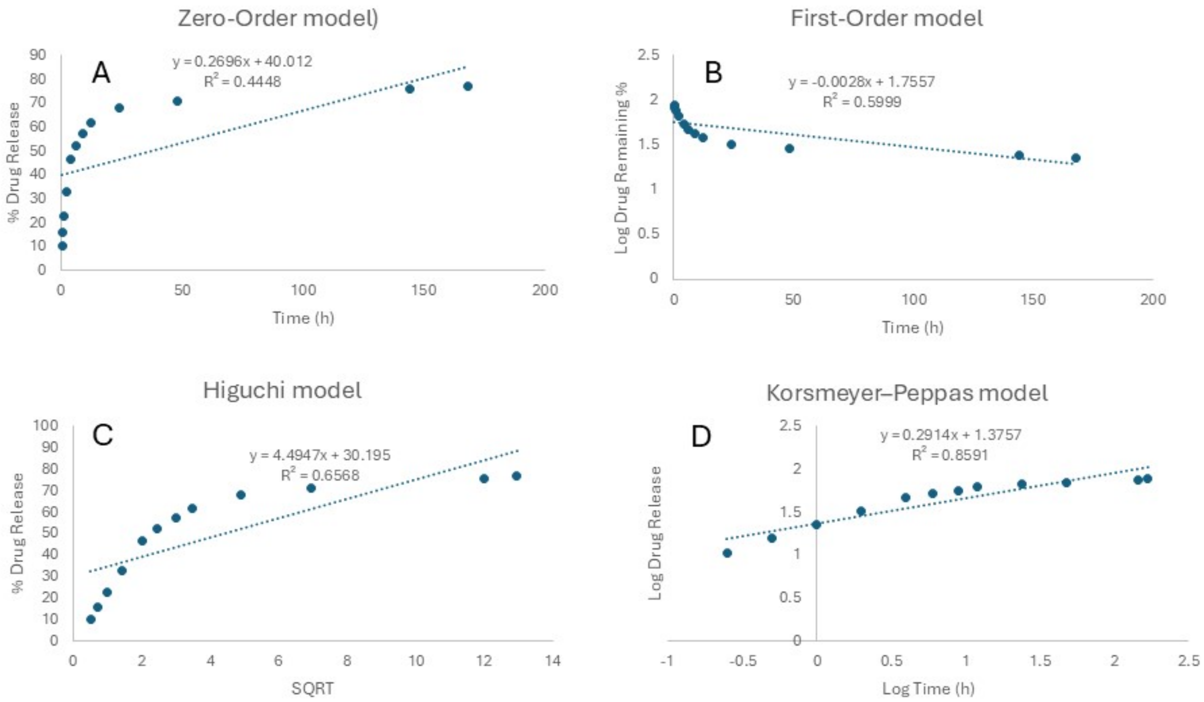

S12. The release kinetics model fitting curves of **RRME-ICs** at pH 5.5: (A) zero-order release kinetics; (B) first-order kinetics model; (C) Higuchi model; and (D) Korsmeyer–Peppas model.

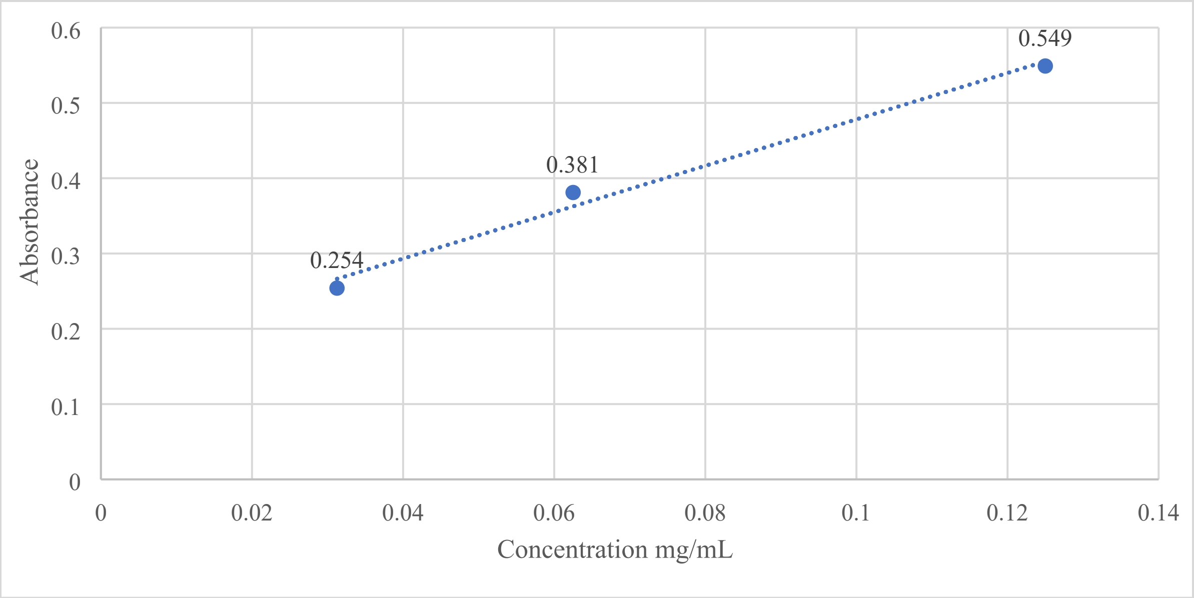

S13. Standard curve for measuring the total phenolic content of the extract using the Folin-Ciocalteu assay.

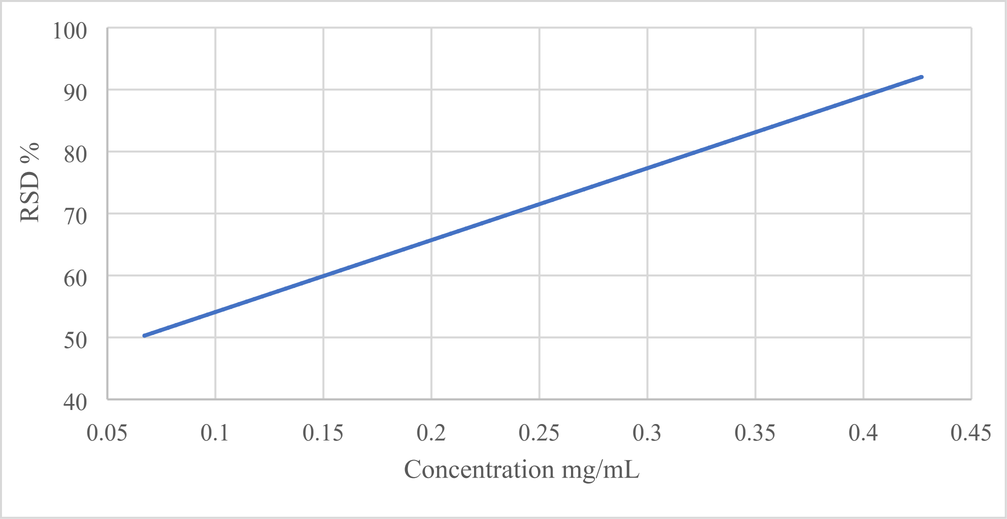

S14. Radical Scavenging assay for RRME.

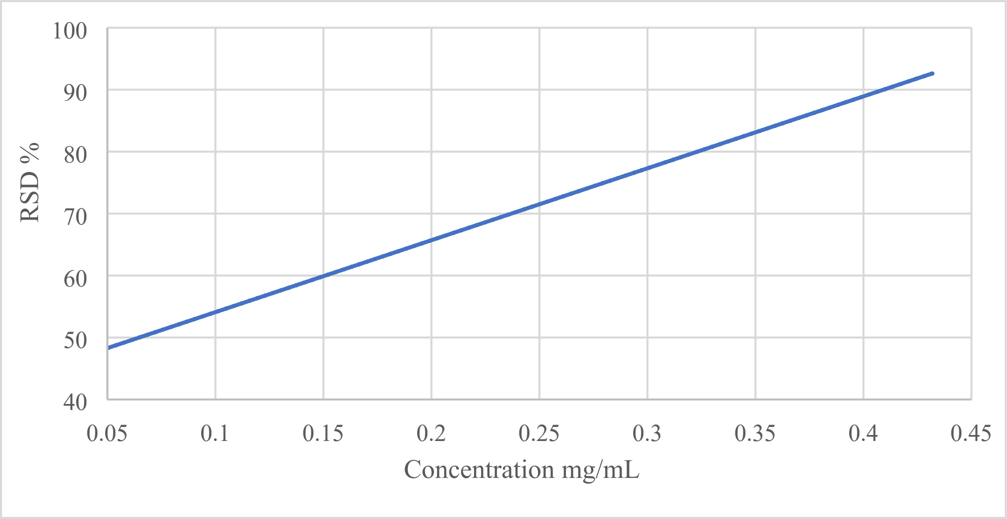

S15. Radical Scavenging assay for RRME-ICs.
